# Effectiveness of Online Collaborative Care for Treating Mood and Anxiety Disorders in Primary Care: A Randomized Clinical Trial

**DOI:** 10.1001/jamapsychiatry.2017.3379

**Published:** 2017-11-08

**Authors:** Bruce L. Rollman, Bea Herbeck Belnap, Kaleab Z. Abebe, Michael B. Spring, Armando J. Rotondi, Scott D. Rothenberger, Jordan F. Karp

**Affiliations:** 1 Division of General Internal Medicine, University of Pittsburgh School of Medicine, Pittsburgh, Pennsylvania; 2 Center for Behavioral Health and Smart Technology, University of Pittsburgh School of Medicine, Pittsburgh, Pennsylvania; 3 School of Information Science and Technology, University of Pittsburgh, Pittsburgh, Pennsylvania; 4 Department of Critical Care Medicine, University of Pittsburgh School of Medicine, Pittsburgh, Pennsylvania; 5 Department of Psychiatry, University of Pittsburgh School of Medicine, Pittsburgh, Pennsylvania; 6 Center for Clinical Trials and Data Coordination, University of Pittsburgh School of Medicine, Pittsburgh, Pennsylvania; 7 Mental Illness Research, Education, and Clinical Center, VA Pittsburgh Health Care System, Department of Veterans Affairs, Pittsburgh, Pennsylvania

## Abstract

**Importance:**

Collaborative care for depression and anxiety is superior to usual care from primary care physicians for these conditions; however, challenges limit its provision in routine practice and at scale. Advances in technology may overcome these barriers but have yet to be tested.

**Objective:**

To examine the effectiveness of combining an internet support group (ISG) with an online computerized cognitive behavioral therapy (CCBT) provided via a collaborative care program for treating depression and anxiety vs CCBT alone and whether providing CCBT in this manner is more effective than usual care.

**Design, Setting, and Participants:**

In this 3-arm randomized clinical trial with blinded outcome assessments, primary care physicians from 26 primary care practices in Pittsburgh, Pennsylvania, referred 2884 patients aged 18 to 75 years in response to an electronic medical record prompt from August 2012 to September 2014. Overall, 704 patients (24.4%) met all eligibility criteria and were randomized to CCBT alone (n = 301), CCBT+ISG (n = 302), or usual care (n = 101). Intent-to-treat analyses were conducted November 2015 to January 2017.

**Interventions:**

Six months of guided access to an 8-session CCBT program provided by care managers who informed primary care physicians of their patients’ progress and promoted patient engagement with our online programs.

**Main Outcomes and Measures:**

Mental health–related quality of life (12-Item Short-Form Health Survey Mental Health Composite Scale) and depression and anxiety symptoms (Patient-Reported Outcomes Measurement Information System) at 6-month follow-up, with treatment durability assessed 6 months later.

**Results:**

Of the 704 randomized patients, 562 patients (79.8%) were female, and the mean (SD) age was 42.7 (14.3) years. A total of 604 patients (85.8%) completed our primary 6-month outcome assessment. At 6-month assessment, 254 of 301 patients (84.4%) receiving CCBT alone started the program (mean [SD] sessions completed, 5.4 [2.8]), and 228 of 302 patients (75.5%) in the CCBT+ISG cohort logged into the ISG at least once, of whom 141 (61.8%) provided 1 or more comments or posts (mean, 10.5; median [range], 3 [1-306]). Patients receiving CCBT+ISG reported similar 6-month improvements in mental health–related quality of life, mood, and anxiety symptoms compared with patients receiving CCBT alone. However, compared with patients receiving usual care, patients in the CCBT alone cohort reported significant 6-month effect size improvements in mood (effect size, 0.31; 95% CI, 0.09-0.53) and anxiety (effect size, 0.26; 95% CI, 0.05-0.48) that persisted 6 months later, and completing more CCBT sessions produced greater effect size improvements in mental health–related quality of life and symptoms.

**Conclusions and Relevance:**

While providing moderated access to an ISG provided no additional benefit over guided CCBT at improving mental health–related quality of life, mood, and anxiety symptoms, guided CCBT alone is more effective than usual care for these conditions.

**Trial Registration:**

clinicaltrials.gov Identifier: NCT01482806

## Key Points

**Questions:**

Is combining an internet support group (ISG) with a care manager–guided computerized cognitive behavioral therapy (CCBT) program better at treating depression and anxiety than CCBT alone and better than primary care physicians’ usual care for these conditions?

**Findings:**

Among 704 patients randomized to CCBT+ISG, CCBT alone, or their primary care physicians’ usual care, patients in the CCBT+ISG and CCBT alone cohorts reported similar improvements in mental health–related quality of life, mood, and anxiety symptoms, while patients in the CCBT alone cohort reported greater improvements in mood and anxiety than usual care.

**Meaning:**

Providing moderated access to ISG provided no measurable benefit at treating depression and anxiety over care manager–supported CCBT; however, care manager–supported CCBT was more effective than primary care physicians’ usual care for these conditions.

## Teaser

This randomized clinical trial examines the effectiveness of combining an internet support group with an online computerized cognitive behavioral therapy provided via a collaborative care program for treating depression and anxiety vs computerized cognitive behavioral therapy alone, and whether providing computerized cognitive behavioral therapy in this manner is more effective than primary care physicians’ usual care.

## Introduction

Dozens of trials have proven the effectiveness of collaborative care strategies at treating mood and anxiety disorders in primary care.^1^ These programs typically involve nonphysician care managers who promote use of evidence-based treatment protocols and monitor patients’ clinical response under the supervision of their primary care physicians (PCPs). However, challenges hinder provision of collaborative care into routine practice and at scale.^2^

Enabled by advances in computer technology, several computerized cognitive behavioral therapy (CCBT) programs have been developed and proven to be as effective as face-to-face therapy at treating depression and anxiety in primary care.^3,4,5^ These programs have the advantages of convenient 24/7 access, avoidance of stigma incurred by seeing a therapist, and greater consistency and scalability compared with traditional therapy. Still, while CCBT has been used by hundreds of thousands of patients in Europe and Australia, it remains largely unknown and little used within the United States.^6^

Another recent development has been the rise of internet support groups (ISGs) that offer general health and disease-specific information and enable members to share treatment information and provide peer support.^7^ Indeed, some ISGs have evolved into large-scale sites with thousands of members organized into numerous disease-specific groups.^8,9^ Yet despite indications of benefit,^9,10,11,12,13^ to our knowledge, their effectiveness has not been firmly established.^14^

Providing patients with depression and anxiety with guided access to CCBT either alone or in combination with an ISG may be an ideal method to deliver effective mental health care at scale. This report presents the main findings from the Online Treatments for Mood and Anxiety Disorders in Primary Care, the first randomized trial to evaluate the effectiveness of providing these technologies through a collaborative care program.

## Methods

### Study Setting

Using a protocol approved by the University of Pittsburgh Institutional Review Board, our single-center trial recruited patients from 26 primary care offices that shared a common electronic medical record (EMR) (Epic). Informed written consent was obtained from all participants. The trial protocol can be found in Supplement 1.

### Participants

We exposed PCPs to an EMR “Best Practice Alert” reminder about our study at the time of the clinical encounter.^15^ It launched automatically for all patients aged 18 to 75 years whenever anxiety, generalized anxiety, panic, or depression was entered as an encounter diagnosis. If the patient agreed to a referral, the PCP electronically “signed” the alert, which forwarded the patient’s name to a study recruiter who then called the patient by telephone to review protocol eligibility.

Eligible patients needed to have internet and email access; a score of 10 or greater on either the 7-Item Generalized Anxiety Disorder scale (GAD-7)^16^ or the 9-Item Patient Health Questionnaire (PHQ-9)^17^; and no alcohol dependence as determined by the Alcohol Use Disorders Identification Test,^18^ active suicidality, or other serious mental illness for which our interventions may be inappropriate. If confirmed, the recruiter reviewed a mailed consent form and obtained the patients’ signed consent on a recorded telephone line. Afterwards, they administered the 12-Item Short-Form Health Survey (SF-12) to determine health-related quality of life,^19^ the fixed-length Patient-Reported Outcomes Measurement Information System (PROMIS) depression and anxiety measures to assess mood and anxiety symptoms,^20^ and the Primary Care Evaluation of Mental Disorders to provide an anxiety and mood disorder diagnosis,^21^ and collected information on patients’ self-reported race/ethnicity, sex, and other sociodemographic characteristics.

### Randomization Procedure

Following the baseline assessment, we randomized patients in a 3:3:1 ratio to (1) care manager–guided CCBT (CCBT alone), (2) care manager–guided access to both CCBT and our ISG (CCBT+ISG), or (3) usual care (UC) under their PCP. We stratified randomization by practice size and age group using randomly permuted blocks according to a computer-generated assignment sequence prepared in advance by our study statistician and concealed until after the baseline assessment. Afterwards, we informed all patients of their treatment assignment and notified their referring PCP.

### Usual Care

For ethical reasons,^22^ we informed patients receiving UC of their mood and anxiety symptoms and their referring PCP. However, we provided no treatment advice unless we detected suicidality or a 25% worsening of symptoms from baseline on a follow-up assessment.

### Interventions

We employed college graduates with mental health research experience as care managers and assigned each exclusively to one intervention arm. We first prepared them in a basic understanding of mood and anxiety disorders, our pharmacotherapy algorithm,^23^ CCBT program, and tracking registry and later reinforced this training in our weekly case review sessions.

### Computerized Cognitive Behavioral Therapy

We used the Beating the Blues CCBT program, which has been proven to be effective.^24,25^ It consists of a 10-minute introductory video followed by eight 50-minute interactive sessions that our care managers encouraged patients to complete every 1 to 2 weeks. Each session used easily understood text, audiovisual clips, and “homework” assignments to impart basic CBT techniques, and patients completed the GAD-7 and PHQ-9 at the start of each CCBT session to self-track their symptoms (eMethods in Supplement 2).

### Internet Support Group

We used WordPress software to create our password-protected ISG that patients could access via computer or smartphone (eFigure 1 in Supplement 2). In addition to a variety of discussion boards created by the care manager moderator and study patients, the ISG curated links to external resources, including local $4 generic pharmacy programs, “find-a-therapist” and various crisis hotlines, and brief YouTube videos on insomnia, nutrition, exercise, and other topics, and we embedded links to our EMR’s patient portal to integrate its use into routine care. To enhance patient engagement, we featured (1) status indicators on members’ profiles and comments (eg, stars and “likes”), (2) email notifications of new ISG activities, (3) automated highlighting of recent comments on members’ home pages personalized to their ISG profile and past activities, (4) invited member-guest moderators, and (5) various contests to encourage log-ins and comments.

To preserve confidentiality, we assigned members’ user names, encouraged them to select a representative avatar (eg, a sunrise or animal), and sent reminders not to post self-identifying information. For additional safety, an investigator logged into the ISG daily to review new posts for suicidal thoughts and other potentially inappropriate content, and we allowed members to flag comments for potential removal.

### Care Manager Contacts

Care managers emailed their assigned patients a web link to the CCBT program and, if applicable, the ISG and requested a time to schedule an introductory telephone call to review the program(s) and establish rapport. Later, they logged into the CCBT program’s clinical helper portal to monitor their patients’ progress (eg, sessions completed, self-reported symptoms, and problems they chose to address), sent personalized feedback and encouragement via email, and contacted patients via telephone who either had not improved or failed to log in regularly.

### Case Review and Follow-up

Care managers presented their patients to the study PCP, psychiatrist, and project coordinator in weekly 60-minute case review sessions split by intervention arm. To efficiently focus our time, we developed an electronic registry that could sort patients by randomization date, last contact, and highest PHQ-9 or GAD-7 score (eMethods in Supplement 2). In addition to conveying general lifestyle adjustments, including exercise and social engagement, we recommended antidepressant/anxiolytic pharmacotherapy based on patients’ treatment preferences and response to CCBT as well as referrals to mental health specialists when they did not improve or had complex psychosocial issues.^23^

Depending on a patient’s symptoms and level of engagement, the care manager emailed or telephoned biweekly for approximately 2 months, and these contacts lasted approximately 15 to 30 minutes. Afterwards, the patient transitioned to the continuation phase of care, during which the care manager contacted the patient approximately monthly until the end of our 6-month intervention. Given our collaborative care framework, we provided PCPs with our treatment recommendations and regular updates of their patients’ progress via EMR.

### Assessments

Patients, PCPs, and care managers were not blinded to their treatment assignment. Therefore, we employed several blinded assessors to determine the effectiveness of our interventions. They contacted patients by telephone to administer our assessment battery at 3-month, 6-month, and 12-month follow-up and later sent patients $15 after each completed assessment for their time (up to $60).

We trained our assessors using audiotapes, manuals, and practice interviews and used a computer-assisted telephone interview system to guide them through each assessment. We digitally recorded these calls and conducted periodic spot checks to confirm responses were rated accurately and corresponded with those entered into our study database, reviewed interactions with suicidal patients, and provided staff with feedback on their performance. Later, we abstracted data from the EMR to collect information on patients’ medical conditions and health services use, our server logs to measure engagement with the CCBT and ISG programs, and our care managers’ electronic registry to document the number of email and telephone contacts.

### Data and Safety Monitoring

We programmed our computer-assisted telephone interview system to identify patients receiving UC whose blinded PROMIS score increased by 25% or more above baseline. Following a review, we notified their PCP via EMR and offered treatment advice. Whenever our care managers or assessors encountered suicidality, either expressed spontaneously or on routine administration of our measures, our computer-assisted telephone interview system automatically launched our Suicide Risk Management Protocol that provided triage advice.^26^ The CCBT program also notified the care manager whenever a patient endorsed suicidality on the PHQ-9 administered at each session. Finally, an independent external data and safety monitoring board appointed by our funding agency monitored the progress and safety of our trial.

### Statistical Analysis

We powered our trial to test the primary hypothesis that patients receiving CCBT+ISG will report 0.30 or greater effect size (ES) improvement from baseline at 6 months on the SF-12 Mental Health Composite Scale (MCS) vs CCBT alone. Assuming a 2-sample *t* test to compare between-arm differences in 6-month improvements and 2-tailed α = .05, we needed 300 patients per arm to have 90% or greater power to detect a 0.30 ES difference in our primary outcome measure.

We compared baseline sociodemographic and clinical characteristics by randomization status using *t* tests for continuous data and χ^2^ analyses for categorical data. Our primary intent-to-treat analyses included all randomized participants regardless of adherence to their assigned treatment. We used linear mixed models^27^ that included fixed effects for time, study arm, time-by-study arm, age strata, practice size, and random effects for patients. We considered time as a categorical variable because of the assumption of nonlinearity over time. To test our hypotheses, we used contrasts to estimate the adjusted mean difference between study arms in the 6-month improvement of SF-12 MCS and PROMIS measures (secondary outcomes) and 6 months later to assess treatment durability. Additionally, we calculated ESs for 6-month changes in SF-12 with 95% CIs by (1) prespecified subgroups of age group, sex, race/ethnicity, baseline symptom severity, and practice size and (2) unplanned subgroups of education and living alone status. We considered a significant 3-way interaction between time, study arm, and the potential covariate as a significant subgroup effect and used Poisson regression to compare rates of PCP contacts, emergency department visits, and hospitalizations between study arms.

We investigated a potential dose response between the number of CCBT sessions completed within our CCBT alone and UC study arms using the same linear mixed model described earlier but parametrizing the CCBT alone arm by assigning each patient a value equal to the proportion completed of the 8-session program. Finally, we conducted exploratory post hoc per-protocol analyses restricted to those who completed 4 or more and all 8 CCBT sessions.

Every effort was made to identify the mechanism of missing data. We compared participants who withdrew from study participation by baseline covariates and analyzed time until withdrawal by study arm using Kaplan-Meier curves.^28^ Our linear mixed models for our primary analyses assumed that data were missing at random and were robust to ignorable missingness assumptions.^29^ All reported *P* values are 2-tailed with significance levels at *P* ≤ .05, and all analyses were performed with SAS version 9.4 (SAS Institute).

## Results

**Figure 1.**
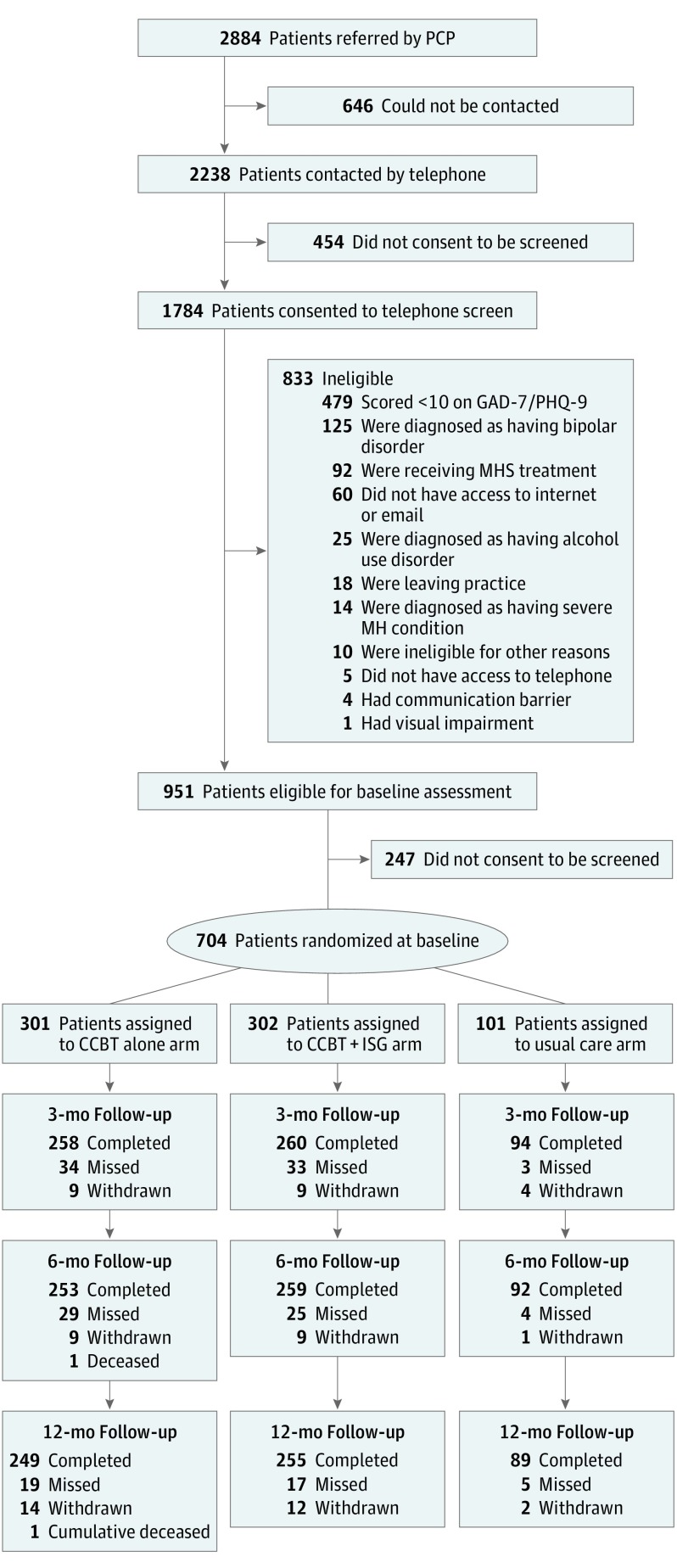
Flowchart of Participants Participants were referred by primary care physicians (PCPs) between August 2012 and September 2014. CCBT indicates computerized cognitive behavioral therapy; GAD-7, 7-Item Generalized Anxiety Disorder Scale; ISG, internet support group; MH, mental health; MHS, mental health specialist; PHQ-9, 9-Item Patient Health Questionnaire.

From August 2012 to September 2014, PCPs referred 2884 patients in response to our EMR prompt. Of these, 704 (24.4%) met all eligibility criteria, provided informed consent, and were randomized (Figure 1). Their baseline sociodemographic and clinical characteristics (Table 1) and completion rate of follow-up assessments at both 6 months (604 [85.8%]) and 12 months (593 [84.2%]) were similar by randomization status (Figure 1), and we found no differences in the sociodemographic and clinical characteristics between participants who withdrew and those who did not.

**Table 1.** Baseline Sociodemographic and Clinical Characteristics by Randomization Status

Characteristic	No. (%)			
	Overall (N = 704)	CCBT alone (n = 301)	CCBT+ISG (n = 302)	Usual Care (n = 101)
Age, mean (SD)	42.7 (14.3)	43.0 (14.0)	42.6 (14.4)	41.7 (14.6)
Age group, y				
18-34	256 (36.4)	108 (35.9)	111 (36.8)	37 (36.6)
35-59	343 (48.7)	149 (49.5)	143 (47.4)	51 (50.5)
60-75	105 (14.9)	44 (14.6)	48 (15.9)	13 (12.9)
Female	562 (79.8)	235 (78.1)	245 (81.1)	82 (81.2)
Race/ethnicity				
White	576 (81.8)	257 (85.4)	242 (80.1)	77 (76.2)
African American	113 (16.5)	38 (12.6)	53 (17.5)	22 (21.8)
Other	15 (2.1)	6 (2.0)	7 (2.3)	2 (2.0)
College degree or higher	333 (47.3)	137 (45.5)	144 (47.7)	52 (51.5)
Married or living with partner	283 (40.2)	123 (40.9)	120 (39.7)	40 (39.6)
Living alone	125 (17.8)	54 (17.9)	60 (19.9)	11 (10.9)
Employed	492 (69.9)	217 (72.1)	204 (67.5)	71 (70.3)
Practice size				
Large (≥6 PCPs)	433 (61.5)	185 (61.5)	186 (61.6)	62 (61.4)
Small (<6 PCPs)	271 (38.5)	116 (38.5)	116 (38.4)	39 (38.6)
Mental health disorder^a^				
Major depression	597 (84.8)	258 (85.7)	257 (85.1)	82 (81.2)
Generalized anxiety disorder	313 (44.5)	135 (44.9)	124 (41.1)	54 (53.5)
Panic disorder	160 (22.7)	65 (21.6)	79 (26.2)	16 (15.8)
Both depression and anxiety	499 (70.9)	219 (72.8)	207 (68.5)	73 (72.3)
Depression/anxiety medication use within past year	544 (77.3)	232 (77.1)	236 (78.1)	76 (75.2)
PHQ-9 score, mean (SD)^b^^,^^c^	13.3 (5)	13.2 (5.3)	13.4 (4.7)	13.1 (4.9)
PHQ-9 score ≥ 15	281 (39.9)	119 (39.5)	122 (40.4)	40 (39.6)
GAD-7 score, mean (SD)^b^^,^^d^	12.9 (4.4)	13.0 (4.3)	12.6 (4.5)	13.5 (4.2)
GAD-7 score ≥ 15	257 (36.5)	114 (37.9)	102 (33.8)	41 (40.6)
PROMIS Depression T-score, mean (SD)^e^	62.1 (6.3)	62.5 (6.2)	62.0 (6.3)	61.4 (6.4)
PROMIS Anxiety T-score, mean (SD)^f^	65.8 (6)	65.9 (6)	65.8 (6.2)	65.4 (5.7)
SF-12 MCS, mean (SD)^g^^,^^h^	31.4 (9)	31.3 (8.4)	31.7 (9.4)	31.1 (9.3)
SF-12 PCS, mean (SD)^g^^,^^h^	51.1 (12.3)	50.7 (12.2)	51.0 (12.3)	52.2 (12.7)

Abbreviations: CCBT, computerized cognitive behavioral therapy; GAD-7, 7-Item Generalized Anxiety Disorder Scale; ISG, internet support group; PCP, primary care physician; PHQ-9, 9-Item Patient Health Questionnaire; PROMIS, Patient-Reported Outcomes Measurement Information System; 12-Item Short-Form Health Survey Mental Health Composite Scale; SF-12 PCS, 12-Item Short-Form Health Survey Physical Health Composite Scale.

^a^ Determined using Primary Care Evaluation of Mental Disorders.

^b^ Higher scores indicate more severe symptoms.

^c^ Range, 0-27.

^d^ Range, 0-21.

^e^ T-score range, 37.1-81.1.

^f^ T-score range, 36.3-82.7.

^g^ Range, 0-100.

^h^ Higher scores indicate better health-related quality of life.

### Intervention Engagement

By 6 months, 504 of 603 patients (83.6%) with CCBT access had completed at least 1 session and 221 (36.7%) had completed all 8, and the mean sessions completed was 5.4, which was similar by randomization status (Table 2), sex, race/ethnicity, and age strata (eTable 1 in Supplement 2). Overall, 228 of 302 patients (75.5%) in the CCBT+ISG arm logged into the ISG at least once, of whom 141 (61.8%) made at least 1 online comment or post (mean, 10.5; median, 3; range, 1-306) (Table 2) (eFigure 2 in Supplement 2).

**Table 2.** 6-Month Care Processes and Health Services Use Following Randomization

Characteristic	CCBT Alone (n = 301)	CCBT+ISG (n = 302)	Usual Care (n = 101)
Beating the Blues CCBT, No. (%)			
Participants who logged in	261 (86.7)	260 (86.1)	NA
CCBT sessions completed of those who completed ≥1 session, mean (SD) [denominator]	5.4 (2.8) [254]	5.5 (2.7) [250]	NA
No. of participants who completed all 8 sessions	112 (37.2)	109 (36.1)	NA
ISG			
Logged in, No. (%)	NA	228 (75.5)	NA
Log-ins per user			
Mean	NA	8.9	NA
Median (range)	NA	4 (1-214)	NA
Commented, No. (%)	NA	138 (45.7)	NA
Comments per commenter			
Mean	NA	9.6	NA
Median (range)	NA	3 (1-285)	NA
Posted, No. (%)	NA	45 (14.9)	NA
Posts per poster			
Mean	NA	3.8	NA
Median (range)	NA	1 (1-42)	NA
Commented or posted, No. (%)	NA	141 (46.7)	NA
Comments/posts per commenter/poster			
Mean	NA	10.5	NA
Median (range)	NA	3 (1-306)	NA
Care management, median (IQR)^a^			
No. of telephone calls	4 (3-6)	3 (2-5)	NA
No. of emails	9 (6-11)	12 (9-16)	NA
No. of total contacts	13 (10-16)	16 (12-20)	NA
Pharmacotherapy, No. (%)^a^			
SSRI/SNRI use at baseline	200 (66.4)	206 (68.2)	66 (65.3)
SSRI/SNRI use at 6 mo, No./total No. (%)	164/253 (64.8)	166/259 (64.1)	50/92 (54)
Benzodiazepine use at baseline	39 (13.0)	40 (13.2)	14 (13.9)
Benzodiazepine use at 6 mo, No./total No. (%)	31/253 (12.3)	29/259 (11.2)	9/92 (10)
Health care use, median (range)^a^			
PCP office visits	2 (0-12)	2 (0-16)	2 (0-7)
PCP telephone contacts	0 (0-7)	0 (0-7)	0 (0-4)
PCP email contacts	0 (0-7)	0 (0-11)	0 (0-6)
PCP total contacts	3 (0-18)	4 (0-28)	3 (0-11)
Mental health specialty visit, No./total No. (%)	45/267 (16.9)	69/271 (25.5)	17/95 (18)
ED visits	0 (0-5)	0 (0-7)	0 (0-4)
Hospitalizations	0 (0-4)	0 (0-2)	0 (0-3)

Abbreviations: CCBT, computerized cognitive behavioral therapy; ED, emergency department; IQR, interquartile range; ISG, internet support group; NA, not applicable; PCP, primary care physician; SSRI, selective serotonin reuptake inhibitor; SNRI, serotonin norepinephrine reuptake inhibitor.

^a^ Data from medical record abstraction.

### Primary Hypothesis: CCBT+ISG vs CCBT Alone

**Figure 2.**
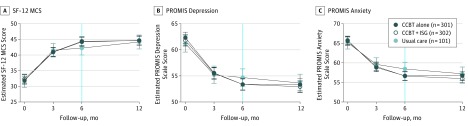
Estimated Scores by Baseline Treatment Assignment Linear mixed models adjusted for time, study arm, time-by-study arm, age strata, and clinic size. A, Estimated scores for the 12-Item Short-Form Health Survey Mental Health Composite Scale (SF-12 MCS). B, Estimated scores for the Patient-Reported Outcomes Measurement Information System (PROMIS) Depression scale. At 6 months, patients receiving computerized cognitive behavioral therapy (CCBT) alone vs usual care reported a −2.43 (95% CI, −4.16 to −0.69; *P* = .006) improvement. C, Estimated scores for the PROMIS Anxiety scale. At 6 months, patients receiving CCBT alone vs usual care reported a −2.30 (95% CI, −4.21 to −0.4; *P* = .02) improvement. The vertical line at 6 months indicates the end of care manager–led CCBT and our primary outcome point. The following 6 months were naturalistic follow-up to observe the durability of our interventions. The error bars indicate 95% CIs. ISG indicates internet support group.

**Figure 3.**
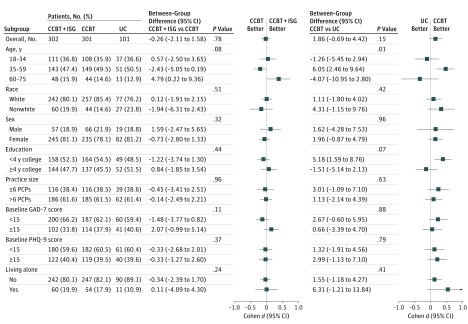
Forest Plot of Between-Group Differences and Effect Sizes for the 12-Item Short-Form Health Survey Mental Health Composite Scale CCBT indicates computerized cognitive behavioral therapy; GAD-7, 7-Item Generalized Anxiety Disorder Scale; ISG, internet support group; PCP, primary care physician; PHQ-9, 9-Item Patient Health Questionnaire; UC, usual care.

At 6-month follow-up, patients in the CCBT+ISG and CCBT alone arms reported similar improvements on our primary outcome measure (SF-12 MCS: ES, 0.02; 95% CI, −0.17 to 0.13) and on the PROMIS Depression and Anxiety scales that continued 6 months later (Figure 2). We also identified a significant treatment interaction favoring CCBT+ISG for patients aged 60 to 75 years on the SF-12 MCS (Figure 3) (eFigure 3 in Supplement 2) and CCBT alone for patients aged 35 to 59 years on the PROMIS Depression and Anxiety scales (eFigure 4 in Supplement 2).

### Secondary Hypothesis: CCBT Alone vs UC

Compared with patients in the UC arm, patients in the CCBT alone arm reported significant 6-month improvements on the PROMIS Depression and Anxiety scales (eFigure 4 in Supplement 2) but not the SF-12 MCS scale (Figure 2). However, these differences resolved 6 months later, as patients’ symptoms in the UC arm improved. Again, we observed significant treatment interactions favoring CCBT for patients aged 35 to 59 years on the SF-12 MCS (eFigure 3 in Supplement 2) and PROMIS Depression and Anxiety scales (eFigure 4 in Supplement 2), for patients living alone on the PROMIS Depression and Anxiety scales, and for nonwhite patients on the PROMIS Depression scale (eFigure 4 in Supplement 2). Moreover, patients reported improved 6-month SF-12 MCS (mean points, 0.80; 95% CI, 0.37-1.22), PROMIS Depression (mean points, 0.48; 95% CI, −0.76 to −0.19), and PROMIS Anxiety (mean points, 0.48; 95% CI, −0.79 to −0.17) scores for each additional CCBT session completed, and per-protocol analyses revealed a similar pattern (PROMIS mood symptoms: patients who completed ≥4 sessions: ES, 0.41; 95% CI, 0.17-0.65; patients who completed all 8 sessions: ES, 0.52; 95% CI, 0.26-0.78) (eTable 2 in Supplement 2).

### 6-Month Health Services Use

Primary care physicians and care manager contacts with patients via telephone and email were similar by intervention arm (Table 2). Moreover, patients receiving UC and intervention had similar rates of PCP contacts, use of antidepressant and anxiolytic pharmacotherapy, and visits to mental health specialists, emergency departments, and hospitals (Table 2).

## Discussion

To our knowledge, this is the first trial to examine the effectiveness of incorporating either CCBT or an ISG into a collaborative care program for treating depression or anxiety in primary care. Our report confirms the effectiveness of guided CCBT, highlights the critical importance of patient engagement with online interventions, and provides high-quality evidence about the limits and potential benefits of these emerging technologies.

Few trials have evaluated the psychologic benefits of ISGs, and none were linked to patients’ usual source of primary care as ours was.^7,30,31,32^ Perhaps most comparable with our trial is the trial by Griffiths et al,^31^ who randomized 478 adults with elevated depressive symptoms to either a moderated ISG, an online psychotherapy program, both interventions, or to a UC control. They too found their combined ISG and online psychotherapy interventions improved mood symptoms vs UC and no benefit from their ISG beyond UC. However, engagement was low, as only 62% of patients in the ISG group logged into the site, only 15% created 1 or more ISG posts, and those assigned to the ISG arm were more likely than patients in the UC arm to miss a blinded telephone assessment (52% vs 34% at 12 months).^31^ These findings as well as other reports^33^ add to our understanding of the challenges in sustaining patient engagement with online interventions to improve clinical outcomes.^34,35^

Unlike our earlier collaborative care trials, where care managers assigned patients homework lessons in printed workbooks,^36,37,38^ the CCBT program enabled our care managers to unobtrusively monitor their patients’ engagement with treatment while providing similarly effective care to a doubled caseload (90 to 100 patients).^36,38^ Although the ES improvements we obtained were smaller than those described in meta-analyses of “supported” CCBT (major depression: ES, 0.78; 95% CI, 0.59-0.96^4^), we identified a dose effect that confirms the importance of patient engagement.^39^ Indeed, Gilbody et al^40^ reported in 2015 no differences in mood symptoms among 691 patients with depression in primary care they randomized to either Beating the Blues or MoodGYM (HealthMed) CCBT programs^41^ or usual care from their PCPs. Similar to our protocol, their study staff contacted patients by telephone to promote use of their programs; however, they did not monitor patients’ symptoms or send recommendations to PCPs as we did, and patient adherence with both CCBT programs was low (median sessions completed, <2).^40^

Given our findings and other recent reports,^3,4,5^ we anticipate more engaging and powerful CCBT programs better tailored to patients’ specific needs, sociodemographic characteristics, medical conditions, and cultural and linguistic preferences that are integrated into the EMR for documentation and billing purposes will become widely deployed over the next decade. Finally, while we were unable to demonstrate a measurable benefit from our ISG, we remain optimistic that more engaging ISGs that apply machine learning algorithms to EMR and claims data to present patients with more personalized information in real time will soon be tested by health care organizations experimenting with social media.^42,43^

### Limitations

Our study has limitations, several of which potentially affect the generalizability of our findings. First, our use of EMR-generated prompts to promote identification of patients for study participation is limited to settings with systems capable of generating these alerts, clinician recognition of targeted conditions, and entry of the proper diagnostic codes into the EMR. Second, we relied on 1 CCBT and ISG, and others using different programs and levels of human support to promote adherence may obtain different outcomes. Third, because we lacked information on symptom duration, patients with long-term mild mood and anxiety symptoms may have similar outcomes as those with severe acute symptoms. Fourth, given the nature of our interventions, patients knew their treatment assignment, which could have biased their responses to our blinded assessors. Finally, study sites were not cluster randomized, and the same physicians cared for patients in all study arms. Although this could have diminished outcome differences between treatment arms, we observed similar ES improvements as previous collaborative care trials.^1^

## Conclusions

In summary, although our ISG did not produce any measurable benefit over CCBT alone, providing online CCBT to patients with depression and anxiety receiving primary care via a centralized collaborative care program is an effective strategy for delivering mental health care at scale. Our study findings have important implications for transforming the way mental health care is delivered in primary care and focus further attention to the emerging field of e–mental health.

## Notes

Supplement 1. Trial protocol.

Supplement 2. **eMethods.** Statistical analysis plan (from funded grant application). **eTable 1.** Computerized cognitive behavior therapy sessions completed at 3 and 6 months following randomization. **eTable 2.** Effect size improvements by number of computerized cognitive behavior therapy (CCBT) sessions completed (CCBT alone vs usual care). **eFigure 1.** Screenshots of internet support group pages. **eFigure 2.** Boxplots of the number of logins, posts, comments, and posts or comments on internet support group. **eFigure 3.** Estimated scores by baseline treatment assignment for the SF-12 MCS by age. **eFigure 4.** Forest plots of between-group differences and effect sizes on the PROMIS Depression (top) and PROMIS Anxiety (bottom) scales.
