# Supplementary material for: Effectiveness of Online Collaborative Care for Treating Mood and Anxiety Disorders in Primary Care: A Randomized Clinical Trial
Source: JAMA Psychiatry. 2017 Nov 8;75(1):56–64. doi: 10.1001/jamapsychiatry.2017.3379 (PMC5833533; doi:10.1001/jamapsychiatry.2017.3379)

eAppendix: Online Treatment Trial for Treating Mood and Anxiety Disorders in Primary Care

Table of Contents

eTable 1. CCBT sessions completed at 3- and 6-months following randomization. 3

eTable 2. Effect size improvements (and 95% CIs) by number of CCBT sessions completed, CCBT-Alone versus Usual Care. 4

eMethods: Trial Protocol 5

1. Research Protocol Abstract 6

2. Recruitment Process (Instructions and conventions) 7

a. Recording Referral (only care managers) 7

b. Entering Referral into Database 7

c. Telephone Call to establish Eligibility (Recruitment) 7

d. Eligibility Forms 7

e. Screening for anxiety severity (GAD-7) 8

f. Screening for mood severity (PHQ-9) 8

g. Mood Diagnosis (PRIME-MD Mood) 8

h. Eligible, Pending or Not Eligible 8

i. Patient Concerns 9

3. Overview of Assessment Process 10

3.1. Baseline Assessments 10

a. Consent Script 10

b. Managing the Return of Consent Forms 10

c. Administration of baseline assessment forms 10

d. Medications for Mental Health History Form 10

e. Family History of Mental Health Conditions 10

f. Drugs, Alcohol, Smoking Protocol & Pharmacotherapy 10

3.2. Follow-Up Assessments 11

a. Administration of forms 11

b. Guidelines for scheduling and contacting assessments 11

c. Reminder calls for schedules appointments 11

3.3. Timeline of Assessment Instruments 12

4. Care Management 13

4.1. Overview 13

4.2. Instructions 13

a. *Newly Randomized Patients* 13
b. *Electronic Registry* 13
c. *Patients with PHQ-9 Scores > 10* 13
d. *Hard to Reach Patients* 14
e. *Withdrawing from Intervention or CCBT Program* 14
f. *End of Intervention* 14
g. *Physician Updates* 14
4.3. Case Review 15
5. *Computerized Cognitive Behavioral Therapy (CCBT)* 16
a. Overview 16
b. *Instructions for Care Manager* 16
6. *Internet Support Group (ISG)* 17
6.1 *Overview* 17
6.2. *Instructions for ISG Moderator* 17
a. *Randomization* 17
b. *First Call* 17
c. *Encouraging ISG Engagement* 18
7. *Suicide Risk Management Protocol* 19
a. *Overview* 19
b. *Administration of SRMP* 19
c. *Contracting for Safety* 19
d. *Resources for suicidal patients* 20
e. *Contacting the on-call psychiatrist* 20
8. *Statistical analysis* 21
a. *Sample Size Calculation* 21
b. *Analysis Sets* 21
c. Handling of Missing Values 21
d. Statistical Analyses 21
Screenshots A. Electronic registry 23
Screenshots B. CCBT program 25
Screenshots C. Internet Support Group (ISG) 30

eTable 1. CCBT sessions completed at 3- and 6-months following randomization.

	At 3 Months*<br>Mean (SD)<br>Completed 1+ sessions<br>% (n)	At 6 Months**<br>Mean (SD)<br>Completed 1+ sessions<br>% (n)
Gender (N)		
Male (124)	4.7 (2.6)<br>80% (99)	5.4 (2.7)<br>81% (101)
Female (479)	4.7 (2.6)<br>83% (396)	5.4 (2.7)<br>84% (403)
Race (N)		
White (499)	4.8 (2.5)<br>84% (421)	5.5 (2.7)<br>86% (429)
Non-White (104)	4.4 (2.7)<br>71% (74)	5.0 (2.7)<br>72% (75)
Age, years (N)		
18-34 (219)	4.6 (2.6)<br>81% (177)	5.3 (2.8)<br>82% (179)
35-59 (292)	4.7 (2.5)<br>82% (238)	5.5 (2.6)<br>84% (244)
60-75 (92)	5.2 (2.8)<br>87% (80)	5.6 (2.9)<br>88% (81)

* Of those who completed ≥ 1 session within the first 3 months following randomization.

** Of those who completed ≥ 1 session within the 6-month intervention period.

eTable 2. Effect size improvements (and 95% CIs) by number of CCBT sessions completed, CCBT-Alone versus Usual Care.

	Intent-to-Treat† ES (95% CI) N=301	≥4 CCBT Sessions Completed ES (95% CI) N=176	All 8 CCBT Sessions Completed ES (95% CI) N=112
SF-12 MCS	0.15 (-0.06 - 0.36)	0.27 (0.05 - 0.49)*	0.42 (0.19 - 0.66)**
PROMIS Depression	0.31 (0.09 - 0.53)*	0.41 (0.17 - 0.65)**	0.52 (0.26 - 0.78)**
PROMIS Anxiety	0.26 (0.05 - 0.48)*	0.34 (0.10 - 0.57)*	0.49 (0.24 - 0.75)**

† - vs. UC, Mixed model, N=402;

* P<0.02,

** P<0.001

eMethods: Trial Protocol

Protocol

1. Research Protocol Abstract

Depression and anxiety are prevalent in primary care practice, associated with substantial reductions in health-related quality of life (HRQoL), and generate a significant excess of morbidity. In response, dozens of trials have demonstrated the greater effectiveness of "Collaborative Care" for these conditions vs. primary care physicians' usual care. Yet for a variety of reasons, these models have not been widely implemented. Therefore, an urgent need remains to develop and test more scalable, powerful, and innovative versions of Collaborative Care while simultaneously developing a greater understanding of how best to provide these interventions through primary care where the majority of depressed and anxious patients seek treatment.

Thousands of web sites provide medical information and the number of Internet support groups (ISG) where the public can exchange information about treatments is proliferating. Still, clinical trials have not established the benefits of utilizing the Internet in this manner. Concurrent with these developments, several computerized cognitive behavioral therapy (CCBT) programs have been proven effective at treating patients with mood and anxiety disorders and used by hundreds of thousands of patients outside the U.S. Yet CCBT remains little utilized inside the U.S., and no trials have incorporated CCBT into a Collaborative Care intervention or examined the effectiveness of combining CCBT with an ISG for these disorders.

We propose a 4-year comparative effectiveness trial that will randomize 700 primary care patients aged 18-75 who have at least a moderate level of mood and/or anxiety symptoms and reliable access to both the Internet and e-mail to either: (1) guided patient access to Beating the Blues, a proven-effective on-line CCBT program (CCBT-alone; N=300); (2) guided patient access to Beating the Blues plus access to a moderated ISG (CCBT+ISG; N=300); or (3) their PCP's "usual care" (N=100). Our primary hypothesis is that patients in our CCBT+ISG arm will report a clinically meaningful 0.30 effect size (ES) or greater improvement in HRQoL on the SF-12 MCS compared to patients in our CCBT-alone arm at 6-months follow-up, and we will monitor patients for an additional 6 months to evaluate the durability of our interventions. Our secondary hypothesis is that CCBT-alone patients will report a 0.50 ES or greater improvement in HRQoL on the SF-12 MCS versus "usual care" at 6-months follow-up. To better understand how online mental health treatments are best provided through primary care, we will also evaluate: (a) their effectiveness across and within age strata; (b) their cost-effectiveness; (c) how patients utilize the components of our interventions; (d) patient subgroups for whom our interventions may be particularly effective; and (e) the adoption and maintenance of our interventions by practices following the Intervention Phase of the Project. Study findings are likely to have profound implications for transforming the way mental health conditions are treated in primary care and focus further attention to the emerging field of e-mental health by other U.S. investigators.

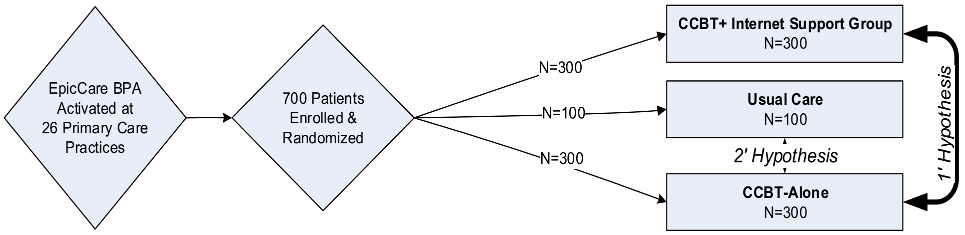

2. Recruitment Process (Instructions and conventions)

a. *Recording Referral (only care managers)*

- Check in-basket in Electronic Medical Record (EMR) system in the morning, early afternoon and after 5 pm for patient referrals (Check BOTH Staff Messages AND BPA Alerts).[1]
- Print out referral and collect it at printer.
- Log out of EMR system
- Give referral to research assessor to enter on database

b. *Entering Referral into Database*

- Open the database and click on "Add potential participant."
- Type in date of referral, physician site, physician and patient initials. Hit submit.
- This will generate a Subject ID. Write down the study ID number to have as a back-up.
- Go to the Recruitment Section and enter demographics as indicated on the referral sheet. Name, gender, DOB, at least one phone number, street name, city, and zip code are required.
- Unless you will be calling patient immediately (unlikely scenario), answer "No" for Question 7, "Reached Patient," Then hit "save for later." Do **NOT** "submit" the form.

c. *Telephone Call to establish Eligibility (Recruitment)*

- Have a paper suicide risk management protocol at hand (see page 17).
- Open database for patient's record, and call patient on phone equipped with digital recording device.
- If patient answers:
  1. Enter "eligibility form"
  2. Follow telephone script
- Are they interested in participating in telephone screen to see if they may qualify?
  1. If no, fill out the reason on form, and thank patient for their time.
  2. If yes, continue.
- Ask permission to audiotape. Begin digital recording. State patient number, date, and time. Ask for permission again, so it is on the recording.
- "I need to ask you some questions about yourself and your medical history in order to see if you are eligible to participate in our study's screening procedure."
- Emphasize that all information is kept confidential on password-protected computers.
- Participation is voluntary.

d. *Eligibility Forms*

- Communication barriers/Telephone access:
  - Ask: "Is this a good number to call if I want to get in touch with you?"
  - If they have substantial trouble hearing you or do not speak English etc. Record as communication barrier
- Access to internet/email.
  - Ask: "Do you have reliable access to the internet?" If no, ask if they can go somewhere on a regular basis to use the internet (library, work, and would feel comfortable to watch a 50-minute video about every 1-2 weeks there)
  - Do you have an email address? Or ask if the one on record is correct.

---

[1] Rollman BL Fischer GS, Zhu F, Herbeck Belnap B. Comparison of electronic physician prompts versus waitroom case-finding on clinical trial enrollment. *J Gen Intern Med* 2008; 23 (4):447-50

- Visual impairment
    - Ask "Do you have trouble seeing content on a computer screen?
- For bipolar disorder questions
    - Make sure these symptoms are a PATTERN of behavior. Everyone goes without sleep or has more energy at particular times. You need to assess whether these symptoms follow a particular pattern for the patient.
    - Assess using provided bi-polar screen.
- Alcohol abuse
    - Ask if they ever drink **any** alcohol, even socially.
    - If they do, make sure to ask them HOW many drinks per week, on average, that they have. If you word it differently and ask, "Do you drink more than 7 drinks per week," they are less likely to give you accurate information. Also probe, asking what they usually drink, wine, beer, or cocktails.
- Substance abuse
    - Ask "Did you ever use recreational drugs (marijuana, cocaine, heroin)?" If yes, ask how often they use these substances, when they tend to use them, when the last time they used them was. If they have used a substance such as marijuana socially and it has been 2 months, this is acceptable to be eligible. However, if they currently use <u>cocaine</u> or <u>heroin</u>, this will definitely be an exclusion.

e. Screening for anxiety severity (GAD-7)

- EMPHASIZE: Over the last 2 weeks….
- REPEAT: "LAST 2 WEEKS" at first 2 questions, and again HALFWAY THROUGH
- REPEAT THE LIKERT SCALE same as above.

f. Screening for mood severity (PHQ-9)

- EMPHASIZE: Over the last 2 weeks….
- REPEAT: "LAST 2 WEEKS" at first 2 questions, and again HALFWAY THROUGH
- REPEAT THE LIKERT SCALE same as above.

g. Mood Diagnosis (PRIME-MD Mood)

- Ask questions relating to the last 2 weeks
- Some questions may be repetitive; please bear with me.
- If applicable, the "Dysthymia" and "Minor Depression options" will drop down; answer them as needed.

h. Eligible, Pending or Not Eligible

- **<u>If eligible:</u>**
    - [Script] "CONGRATULATIONS! You are eligible to participate in our study! We will be mailing you two consent forms to learn more about our study and an answer guide to use during our calls. We will call back in 2-4 days to go over the consent form and answer your questions, ask more questions about anxiety/mood symptoms and your medical history. It will take about half an hour. Is this okay?"
    - Schedule 2-4 business days after today for baseline assessment
- **<u>If Ineligible:</u>**
    - [Script] "Thank you very much for your time, however at this time <your symptoms are not severe enough to be> eligible to continue in our study. Since you have been experiencing some [anxiety/mood] symptoms, we will notify Dr. <*Physician*> who

referred you to our study, and we encourage you to discuss our screening results with him/her. Do you have any questions at this point? Again, please follow-up with your physician. Also, if you think your symptoms will increase at any time, you can ask your physician to refer you again to our study. We will send you a notification via email.

- **Documentation:**
  - In EMR system, write a message to the referring MD (you may look at examples in the shared drive, and ask Project Coordinator what should be included). Let the referring MD know of their patient's ineligibility and reason for ineligibility.

i. Patient Concerns

- *Patient is Concerned about Confidentiality*
  - Reassure patient of their concerns. Explain to them that they are not yet enrolled in the study.
  - Reiterate data safety plan that all information is kept in locked filing cabinets and computers and their data is associated with a 6-digit study id.
  - Explain how we will communicate with their physician should patient be randomized
- *Referred Patients Seeing Mental Health Therapist*
  - Investigate how often the patient is seeing their therapist and what type of treatment they are currently in.
  - If patient is currently in therapy for depression or anxiety symptoms- explain that this no longer makes the patient eligible, as we do not want our intervention to interfere with their current treatment plan.
- *Suicidal Risk Management Protocol*
  - If the patient indicates any level of suicidal ideation during the PHQ-9 screening, or mentions it over the phone, begin the suicide protocol and take appropriate actions
  - See **#6 (page 17)** for more details regarding the suicide risk management protocol.

3. Overview of Assessment Process

3.1. Baseline Assessments

a. Consent Script

- After eligibility is determined, Research Assessor will send patient two copies of the consent form to be reviewed prior to the baseline assessment 2-3 days later
- At the time of the baseline, Research Assessor will review the consent form with patient, and ask for any questions regarding the consent form. This will be done digitally recorded. If the patient consents to participate in trial, s/he will mail consent forms to OT office.
- **Follow Consent Script during call**

b. Managing the Return of Consent Forms

- Signed Consent files are kept in a locked filing cabinet in patient's corresponding folder with any *WePay* receipts.
- Record keeping of all signed consent forms is kept in the Patient Consent Tracker, stored on the shared drive
- Dates consents were sent and returned are kept in the Patient Consent Tracker
- Patients are reminded by phone by Research Assessors and Care Managers to return their consent forms

c. Administration of baseline assessment forms

- Once patient has given consent, the CATI system will guide Research Assessor through administration of baseline forms
- Direct the patient at beginning of each new form to the "answering guide." But repeat the Likert Scale at least once at beginning of administration.
- If patient does not have answering guide at hand, the answering scale has to be repeated every 3-4 questions to ensure that patient is aware of all answering possibilities
- All forms are designed that patient can answer them without interviewer explanations (self-report instruments). If a patient does not understand a question, it has to be repeated as written. DO NOT EXPLAIN THE QUESTION.

d. Medications for Mental Health History Form

- If patient is taking an antidepressant, anti-anxiety medication, benzodiazepines or any other psychiatric medication it MUST be listed even if they person doesn't necessarily take it for Depression/Anxiety.

e. Family History of Mental Health Conditions

- This question asks for patient's family members who are blood/genetic relatives. Also, do not include relatives too far out on the family tree (i.e. do not include dad's cousin's son). Patients with family histories of mental illness are treated differently during care management so it is important to take care in filling out this section at baseline.

f. Drugs, Alcohol, Smoking Protocol & Pharmacotherapy

- If patient states that they are or have been using recreational drugs, it is helpful to ask some follow-up questions to probe more specifically if the patient is still eligible.
- If patient is smoking marijuana- ask how often and in what situations. In most scenarios the patient will still be eligible

- If alcohol use, re-administer questions from screening
- If the patient is on anxiolytics, make specific reference to the particular drug (ie. Valium, Xanax etc.)

3.2. Follow-Up Assessments

a. Administration of forms

- Forms will be pulled up automatically by CATI system. Instructions are same as for baseline assessment (see 3.1.)

b. Guidelines for scheduling and contacting assessments

- **Schedule Appointment before due date:**
    - **1 week before** due date: Send email or EMR message using script reminding of appointment
    - **Every day until** due date: Call once per day until you reach patient to schedule appointment
    - **I**f patient preferred text messages, send a text every 3 days until you reach patient to schedule appointment
- **Reach patient after due date:**
    - **Every day after** due date: Call three times per day, morning, afternoon, evening. Leave message on voice mail every other day.
    - **1 week past** due date: Send second email/EMR message. Continue with above call schedule
    - **2 weeks past** due date, if the patient can still not be contacted: Send a scheduling postcard with letter.
    - **4 weeks past** due date, record assessment as "missed" in data base

c. Reminder calls for schedules appointments

- To know who to call for their appointment reminder, go to database **Main Menu > Visit Scheduling,** search **by Scheduled Date**
- Attempt to reach patient or leave message, stating day and time of scheduled appointment and call back number
- If attempting to remind a patient for an appointment the upcoming date, click "yes" under reminders

3.3. Timeline of Assessment Instruments

Form Number	Instrument	Recruitment	Baseline	3 months	6 months	12 months
1	Demographics	X				
2	Eligibility	X				
3	GAD-7	X				
4	PHQ-9	X				
5	PRIME-MD Anxiety	X				
6	PRIME-MD Mood	X				
7	Consent	X	X			
8	SF-12		X	X	X	X
9	Social Support		X			
10	PROMIS Scales		X	X	X	X
11	Co-morbid Anxiety		X			
12	Usual Activities		X			
13	Usual Activities f/u			X	X	X
14	Mental Health History		X			
15	Mental Health f/u			X	X	X
16	Utilization		X			
17	Utilization f/u			X	X	X
18	PEW		X			
19/20	Sociodemographics		X			
22	LOT Optimism		X		X	
23	Suicidal Ideation Protocol		(X)	(X)	(X)	(X)
32	Chart abstractions (A, B, C, D)	Completed on all patients for the 1 year prior and 1 year following enrollment				
33	ISG Survey				X	
34	BtB Surveys				X	

4. Care Management

4.1. Overview

- Non-behavioral health specialists serve as our care managers to increase the generalizability of our methods to typical practice settings.
- As described previously,[2] patients can choose – in addition to their assigned treatment (CCBT-alone or CCBT+ISG) any combination of the following treatment components: (1) a trial of anxiolytic pharmacotherapy, primarily a selective serotonin reuptake inhibitor or serotonin-norepinephrine reuptake inhibitor, selected by patient preference, prior use, insurance coverage, and adjusted per patient response; or (2) referral to a community mental health specialist in keeping with the patient's insurance coverage.
- Care managers also recommend general lifestyle adjustments including promotion of social engagement, exercise, adequate sleep, reducing alcohol consumption, and quitting tobacco use.

4.2. Instructions

a. Newly Randomized Patients

- Newly randomized patients are notified of their group assignment by the Care Manager within two days of randomization via email or EMR notification, [The Project Coordinator notifies Usual Care patients and their PCP] and asks for a telephone appointment
- During the first telephone call the Care Manager explains all intervention elements to patent and conducts a basic mental health assessment.
- All care manager calls are digitally recorded, unless the patient objects.

b. Electronic Registry

- All care management contacts (via telephone or email) are recorded directly into the electronic registry (see screenshots APPENDIX A).
- The Care manager also enters into registry:
  - Beating the Blues lesson completed
  - PHQ-9/GAD-7 scores entered by patient into Beating the Blues
  - Updated antidepressant/anxiolytic medications and doses
  - Referrals to community mental health specialist
  - Concerns, side effects, and other pertinent information

c. Patients with PHQ-9 Scores > 10

- Patients with PHQ-9 scores greater than 10 should be contacted intervention every 2-3 weeks depending on how they are progressing with their Beating the Blues sessions.
- If a patient has severe symptoms (>15) and potential suicidality, the Care Manager attempts to contact him/her **weekly** until symptoms start to resolve.
- Patients whose symptoms improve over the course of intervention, and fall below a PHQ-9 score of 10, will continue to be called by Care Manager every 3-4 weeks, in addition to emails regarding completed CCBT sessions.

---

[2] Rollman BL, Herbeck Belnap B, Reynolds CF, Schulberg HC, Shear MK. A contemporary protocol to assist primary care physicians in the treatment of panic and generalized anxiety disorders. *Gen Hosp Psychiatry* 2003; 25:74-82

d. Hard to Reach Patients

- If a patient misses an intervention call needs to be contacted because of a high self-recorded PHQ-9/GAD-7 score, the Care Manager will try to contact the patient via telephone, email and/or EMR message following the schedule outlines in 3.2.

e. Withdrawing from Intervention or CCBT Program

- If a patient expresses the wish to withdraw from intervention, the Care manager will use motivational interviewing to understand why the patient wants to withdraw. Possible suggestions are:
  - Fewer contacts
  - Intervention "holiday" (e.g., one month without contacts)
  - Referral to a mental health specialist
  - Assistance to find community groups
  - Consultation with a study clinician
  - Advocacy with the patient's PCP
  - Continue only with blinded assessments
- If a patient feels that they were doing better, or they do not want to continue with the CCBT program, the Care Manager can suggest:
  - Assistance with the program
  - CCBT "holiday" (up to 1 month)
  - Just care manager calls. Then the Care Manager administers PHQ-9/GAD-7 at each call. The patient will still be presented during case review session, and recommendations are still conveyed to PCP.

f. End of Intervention

- At the end of 6-months of study intervention, Care Managers present the patient in case review for final recommendations from the study clinicians.
- The final recommendations are discussed with the patient over the telephone during the final intervention call and also summarized in a letter sent to the patient.
- A final letter is also routed to the patient's PCP in *EMR system,* summarizing the patients change in symptoms during the intervention phase and any final recommendations (templates are available).

g. Physician Updates

- Patients' PCPs are updated about the progress of their patients' mood and anxiety symptoms once monthly, during the 6th month course of intervention (5 updates total and one final recommendation (4.2.f). The care manager reports also entail:
  - Antidepressant/anxiolytic medications the patient reports taking and dosage. If this is different from the prescription, this has to be stated explicitly.
  - Most recent self-reported PHQ-9/GAD-7 scores, with a severity classification and change from last report
- Updates are routed through EMR system with the subject line "Update: Mood and Anxiety Trial".

4.3. Case Review

- Once a week, Care Managers meet separately for each intervention arm with the study team (study PCP, study psychiatrist, study psychologist/project coordinator). Meetings last about one hour.
- Using the electronic registry projected on the wall, the Care Manager can sort their care load by
  - Date of randomization
  - Date of last contact
  - PHQ-9 score
  - GAD-7 score
  - Number of BtB sessions completed
- Patients will be discussed by highest symptom score, most recent contact, and any patient the care manager wants to discuss. Once a month all patients are discussed and those who will reach their endo fo intervention.
- The Care Manager presents each patient by presenting all registry tabs (see **Appendix A**) and adding any details from patient contacts as relevant (e.g., specific stressors, patient concerns, medication side effects)
- The study team then makes treatment recommendations. These can include:
  - review of specific CBT techniques
  - medication adjustments
  - referral to mental health specialists
  - referral to community resources
  - lifestyle changes
  - ISG resources [for CCBT+ISG patients only]
- Whenever a recommendation is formulated, the Care Manager discusses this with the patient.
- For medication adjustments:
  - If the patient is in agreement, the Care manager contacts patient's PCP via *EMR system* if s/he agrees, will prescribe the medication.
  - Care Manager follows up with patient after prescription and again 3-5 days after starting the medication to assess for any side effects.

5. *Computerized Cognitive Behavioral Therapy (CCBT)*

a. Overview

- We use the proven-effective "*Beating the Blues*" (*BtB*) CCBT program.[3]
- It consists of a brief 10-minute introductory video followed by eight 50-minute long interactive sessions (see **Appendix B**).
- Each session utilizes easily understood text, audio, and audiovisual clips and "homework" assignments to impart basic CBT techniques
- Patients enter three problems they wanted to work on over the course of the program
- At the start of each session patients complete GAD-7 and PHQ-9 to self-track their symptoms.
- Care managers can monitor the symptom scores, patient-rated progress of distress experienced by identified problems, and progress in sessions completed. However, the Care manager cannot view the examples and homework the patient enters into the system.
- The system will alert the Care Manager via email if a patient indicates in the BtB program that s/he experiences suicidal ideation.
    - Proceed with the SRMP (7.0)

b. Instructions for Care Manager

- After randomization, the Care Manager emails the patient
    - their group assignment,
    - link to website to BtB
    - activation code for BtB with brief instruction how to log-in
    - request to watch the introductory video
- During first care management call (also see 4.2.a) the Care Manager will discuss/explain the BtB program and trouble shoot any technical problems.
- Care Manager and patient agree on a schedule to complete the sessions. Recommended are every 1-2 weeks. However, the patient can adjust the schedule according to their schedules (e.g., take a break during vacation).
    - **Note:** It is <u>not</u> recommended to complete more than 1 session/ week
- After each completed session, the Care Manager send a brief email to patient, congratulating them on completing another session.
- Care Manager then enters the PHQ-9/GAD-7 scores from the BtB program into the electronic registry
- If a patient has not completed a new session after 2 weeks, Care Manager emails a reminder to patient to start the new session.
- In case that patient does not start another session within one week reminder is sent, Care manager emails patient and
    - Outline content of next session
    - Offer help with technical issues
    - Offer telephone call to discuss any concerns.
- If patient does not respond, follow instructions under 3.2.b.

6. *Internet Support Group (ISG)*

6.1 *Overview*

- Patients randomized to the CCBT + ISG arm have access to an password-protected, moderated Internet Support Group, in addition to the CCBT program "Beating the Blues" (see 5.)
- The ISG provides a "safe space" for our study participants in which they can participate – in privacy - in various discussion boards about managing depression, anxiety, stress, and other related issues with other study participants. Here, study participants can share their own stories, experiences, tips, and advice.
- The ISG also features helpful resources, such as information on discounted pharmacy programs, links to psychotherapy sites, healthy sleep tips, etc.
- The ISG is monitored by a *Moderator*, the Care Manager who is also responsible for patients randomized the CCBT+ ISG arm (see tasks under 4. and 5.).
- In addition to regularly monitoring the depression and anxiety symptoms of patients as *Moderator has following additional tasks:*
  - encourage participants to engage with the discussion boards
  - point to resources available on ISG as indicated
  - create new discussion
  - daily monitor all newly posted content

6.2. *Instructions for ISG Moderator*

a. Randomization

- The Care manager informs all patients arm via e-mail of
  - their group assignment,
  - link to website to BtB
  - activation code for BtB with brief instruction how to log-in
  - request to watch the introductory video
  - link to ISG
  - username and temporary password to log into the ISG
- *Moderator* assigns the username consisting of the first name of the patient, along with the first four digits of their DOB. For example, Chris, born on November 24 would be assigned the username **Chris1124**.
- The patient's temporary password is the last 4 digits of their SSN.
- **Note:** Patients are informed that they should change their password. If they prefer then can also request a different username (especially if they have a distinctive first name).

b. First Call

- During their first intervention call, *Moderator* reiterates the purpose of the ISG and reminds patients that their log-in information is within the first e-mail that was sent to them that contained information about their study assignment. Moderator also offers to help with the first log-in and reminds patients for the confidentiality requirements. Finally, they also review the patient profile on the ISG and encourage the patient to add some information and choose and avatar.
- *Moderator also* encourages patients to log into ISG on a regular basis to look at resources, read new discussion boards, respond and provide support to others by leaving comments on discussion boards, and start their own discussions/posts with concerns, information, or solutions they want to share with the community.

c. Encouraging ISG Engagement

- It is essential to the success of an ISG that patient engage in the ISG. Therefore, a major task of Moderator is to encourage patients to log into the ISG. We used several strategies:

1. Discussion of the week

- Each week a new discussion board (post) is posted by Moderator. The study team discussed possible contents and Moderator then creates the new post. Typical contents are related to explaining and managing depression and anxiety, and related issues such as sleep problems. Also, general problems like work stress, family problems, financial issues are discussed.

2. *Responding to patient posts/comments*

- Moderator monitors the ISG throughout each day. If a patient post/comment remains without respond from another patient for 1-2 business days, then the Moderator comments to ensure that some feedback is given.
- If patient posts/comments and need **immediate attention** (e.g., patient endorsing S/I, breach in confidentiality), the Moderator hides the post/comment and then contacts the patient directly to speak with the patient in more detail regarding their posted comment and determine if further action needed to be taken. After the post is discussed and there is no harm involved or breach in confidentiality, it can be made "visible" again, and then *Moderator* writes an appropriate response. **Note**: In case of suicidality, follow the SRMP (see 7.)

3. Care management contacts

- *During each* ***care management contact, Moderator will*** reference ISG discussions, content, and resources as relevant and encourage patients to log in to view the content. For example, if a patient endorses difficulty sleeping, *Moderator* can discuss the sleep hygiene resources/discussions available on the ISG with the patient. Alternatively, if a patient has difficulty with work stress, *Moderator* can send a link to an ISG discussion on managing work-related stress and/or encourage patient to post their specific concerns for others to comment on.

4. ISG Content E-mails

- *Moderator* sends out weekly e-mails to all CCBT+ ISG participants, pointing to new, discussion or resources on the ISG. These e-mails are sent approximately 2 times/week. Patients can opt out of the 2/week emails, and request just one email per week during the intervention period.

7. *Suicide Risk Management Protocol*

a. *Overview*

- The Suicidal Risk Management Protocol (SRMP)[3] is utilized in the Online Treatment Trial to assess levels of suicidality risk in our study patients. Both Research Assessors and Care Managers use the SRMP.
- Many people who are experiencing depression may feel that life is not worth living anymore. While this is a common feeling to experience (especially given stressful circumstances these patients are in), the SRMP protocol is used to assess the patient's risk of self-harm. The SRMP will help identify patients at-risk and provide triage advice.
- The SRMP also serves as a documentation when talking to mental health specialist or following up with physicians.

b. *Administration of SRMP*

- **Research Assessors.** The CATI system is programmed so that the SRMP is automatically launched at baseline, 3-Month, 6-Month, or 12-Month assessments. Various assessment forms specifically probe for suicidal ideation (eg., PHQ-9, PROMIS, and Prime-MD) and if endorsed will trigger the SRMP launch.
- **Care Managers**. When a patient endorses suicidality on the PHQ-9 administered during a care management call, then the care manager can manually launch the SRMP. Also, if the care manager receives a notification of a patient's suicidality by the BtB program, they should call the patients and launch the SRMP.
- **Manual Launch**. If, patient endorses suicidality spontaneously or online the SRMP can be initiated **any time** by clicking on *As Needed Forms* on the patient homepage.
- The SRMP is designed so that with a minimum of questions the assessor/care manager can get triage advice. Please follow the instructions.
- **Note:** If you feel that the patient may not answer the questions in full, you can always administer more parts of the SRMP.
- After **every** call you complete with a patient that endorses **any** level of suicidal ideation, you should inform the project coordinator.
- **Follow-up**. In the SRMP documentation all discussions with psychiatrist and/or physician should be charted. Then the patient is followed-up with 1-, 3-, 7-, and 30-days later to ensure that treatment recommendations are followed and the patient is not at-risk anymore. **Note:** If the patient endorses suicidality during last call, the SRMP should be restarted.

c. *Contracting for Safety*

- The SRMP may prompt you to contract for safety. This is an agreement between the patient and the health professional that the patient agrees not to take any action to harm themselves within a specified period of time. This will give you time to contact the psychiatrist to get further advice
- **Script:** "I want to follow up with one of our study doctors to see if they have any recommendations to try to help. If I speak with them, can you promise me that you will not take any action to harm yourself until I follow up with you in (insert specific time, such as 1 hour, 2 hours, 3:00pm, etc.)?"

---

[3] Herbeck Belnap B, Schulberg HC, He F, Mazumdar S, Reynolds CF, Rollman BL. Electronic protocol for suicide risk management in research participants. *Journal of Psychosomatic Research* 2015; 78:340-5.

d. ***Resources for suicidal patients***

- *The SRMP may also prompt you to provide resources. We usually give patients the* Re:solve Crisis network number. However, this may be modified, e.g. when patient lives in an area with access to other resources.
- The Re: solve Crisis network provides confidential telephone counseling as well as in-person counseling. It is free of charge and available 24/7.
- **Script:** "If you have these feelings of self-harm again or feelings of hopelessness, I encourage you to contact the Re: solve network. Re: solve provides confidential telephone counseling as well as in-person counseling. It is free of charge and available 24/7. Their information is: 1-888-796 – 8226 (or 1-888-7-YOU-CAN)."

e. ***Contacting the on-call psychiatrist***

- The SRMP will prompt you to contact the on-call psychiatrist, when a patient endorses a **moderate** or **high** level of risk.
- What information do I have to give the on-call psychiatrist?
- **Research Assessors. Due to the study blind, r**esearch Assessors will not be able to provide a detailed background on the patient. They should provide the on- call psychiatrist with the information gathered on the SRMP, with esp. emphasis on the patient's risk factors. They should also provide the on-call psychiatrist with the patient's name, date of birth, and telephone number so they can review the patient's medical record and contact them directly, if necessary. The Project Coordinator will then assign a study team member who is unblinded (e.g., care manager) to cpmplete the SRMP documentation and follow-ups.
- **Care Managers.** In addition to above, the care managers can provide the on-call psychiatrist with more detailed information about the patient's mental health history, current medication, etc. The psychiatrist may then either contact the patient directly to discuss treatment options, or instruct you of the next steps to take with the patient. Alternatively, the Project Coordinator may also assign a patient to you who endorsed suicidality during a blinded assessment.

8. *Statistical analysis*

a. *Sample Size Calculation*

Power for this trial will be based on the primary hypothesis that CCBT+ISG patients will report a clinically meaningful 0.30 6-month effect size (Cohen's d ES) improvement on the SF-12 mental component score (MCS), our primary outcome measure, versus CCBT-alone. Assuming a 90% 6-month assessment completion rate and 2-tailed type I error of 0.05, randomizing 300 subjects per arm (600 total) would provide 90% power to detect a 0.30 ES improvement of CCBT+ISG over CCBT-alone (primary hypothesis) and 0.50 ES improvement of CCBT-alone over UC (secondary hypothesis) on the SF-12 MCS or our other continuous outcome measures (e.g., PROMIS Mood). As patient engagement and response to our interventions could differ by age, we plan to test our primary hypothesis within three age strata: 18-34, 35-59, and 60-75. Assuming our smallest age strata is 25% of our study cohort (60-75) and our other assumptions, then randomiz-ing 150 patients would provide 80% power to detect an ITT ES ≥ 0.50 on our continuous measures within our smallest age strata. Applying these assumptions to our secondary hypotheses (CCBT-alone versus UC), we need to randomize 100 UC patients to have 80% power to detect a 0.70 ES improvement on the SF-12 MCS within our smallest age strata.

b. *Analysis Sets*

- The full analysis set will be based on an intention-to-treat (ITT) analysis, which will comprise all participants who have been randomized to any of the 3 study arms, regardless of length of follow-up or actual intervention received. As an exploratory measure, we will conduct per-protocol (PP) analyses for the secondary hypothesis using various definitions based on the number of CCBT sessions. These PP analysis sets will be based on those definitions (i.e. including participants with all 8 sessions completed).
- The primary outcome is mental health-related quality of life (HRQoL) as defined by the SF-12 Mental Component Score (MCS). Secondary outcomes include mood and anxiety, as measured by the PROMIS Depression and Anxiety scales.

c. Handling of Missing Values

- As a preventive measure, we will make every attempt to document all reasons for missing data. In addition, baseline characteristics will be compared between participants who do and do not withdraw from the study as a way to assess the impact of missing information and attrition. We will also compare the rates of lost-to-follow-up (LTF) between study arms.
- We will investigate the reasons for intermittently missing data (misses an assessment but comes back) and dropouts, and use a likelihood-based or multiple imputation-based procedure if "missing at random" (MAR) is confirmed or consider selection models with a logistic dropout process if the missingness is found to be nonignorable (missing not at random (MNAR)).

d. Statistical Analyses

- Demographic and baseline characteristics will be presented as mean and standard deviations for continuous variables and sample proportions for categorical variables. All descriptive statistics will be accompanied by 95% confidence intervals. Baseline comparisons between study arms (for each hypothesis) will be performed using t-tests for continuous variables, chi-squared tests for categorical variables, or their nonparametric counterparts.
- The primary analyses will consider an intention-to-treat (ITT) analysis with exploratory per-protocol (PP) analyses to supplement the main findings. The ITT and PP analysis sets have been defined previously.

- Primary Outcome
  The ITT analyses will assess the effect of CCBT+ISG (versus CCBT alone) and CCBT alone (versus Usual Care) on 6-month improvements in mental health-related quality of life (HRQoL), as measured by the SF-12 MCS. We will fit a Laird and Ware linear mixed model[16] as a function of the following predictors: study arm, time, time-by-study arm, age group (18-34, 35-64, and 65+), and clinic size (small or large as defined by a size of 6 PCPs). In addition, the intercept will be allowed to vary randomly to account for subject-level variability of the outcome at baseline. The primary hypothesis will involve contrasts to estimate the adjusted mean difference in 6-month improvement on SF-12 MCS between CCBT+ISG and CCBT alone study arms. The secondary hypothesis will be similar, but between CCBT alone and Usual Care study arms.
- Secondary Outcomes
  Similar to SF-12 MCS, a Laird and Ware linear mixed model[16] will be fit to estimate the adjusted mean differences in 6-month improvement on PROMIS-Depression and PROMIS-Anxiety.
- Subgroup and Exploratory Analyses
  Planned subgroup analyses will be conducted for the primary outcome of SF-12 MCS as well as secondary outcomes. A formal hypothesis will be tested with an interaction between each of the study arms and the following subgroups: 1) age group (18-34, 35-59, and 60-75), 2) gender, 3) race (white versus non-white), 4) baseline symptom severity (GAD-7 <15 vs >=15 and PHQ-9 <15 versus >=15), and 5) clinic size (small versus large). A significant 3-way interaction between time, study arm, and the potential covariate will indicate a subgroup effect.
  In order to investigate a potential "dose-response" in the secondary hypothesis (CCBT-Alone versus Usual Care), we will assess the effect of the number of completed BtB sessions has on the primary and secondary outcomes. We will use the same linear mixed model mentioned above, but will parametrize the study arm based on the number of sessions completed. For example, instead of 1 and 0 for CCBT-Alone and Usual Care, respectively, participants in the CCBT-Alone arm will receive a value equal to the proportion of 8 sessions completed. Those who completed none as well as those in the Usual Care arm will receive a value of 0.

Screenshots A. Electronic registry

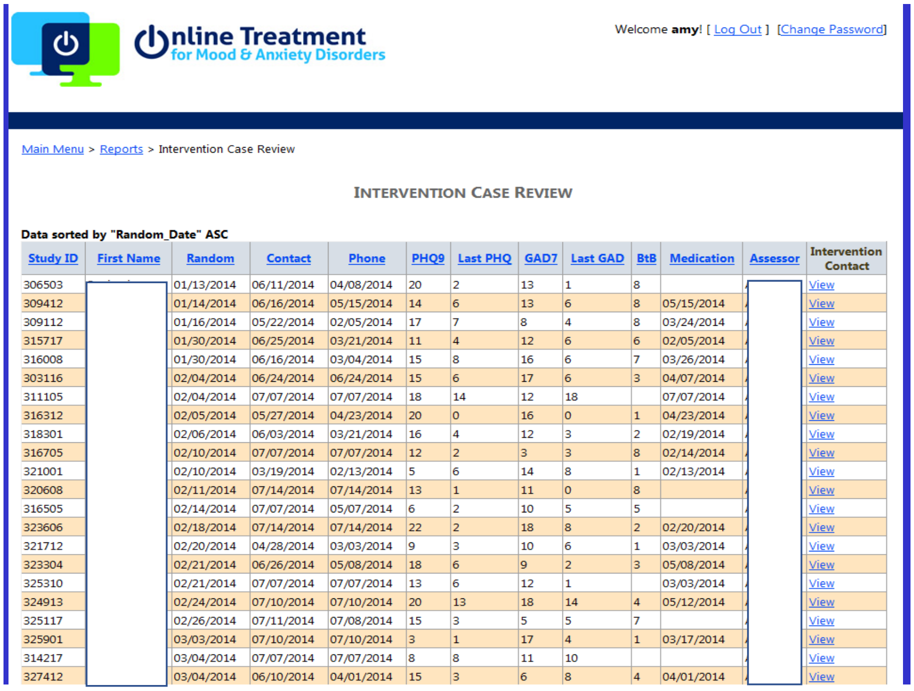

Online Treatment for Mood & Anxiety Disorders

Welcome **amy**! [Log Out] [Change Password]

Main Menu > Reports > Intervention Case Review

INTERVENTION CASE REVIEW

Data sorted by "Random_Date" ASC

Study ID	First Name	Random	Contact	Phone	PHQ9	Last PHQ	GAD7	Last GAD	BtB	Medication	Assessor	Intervention Contact
306503		01/13/2014	06/11/2014	04/08/2014	20	2	13	1	8			View
309412		01/14/2014	06/16/2014	05/15/2014	14	6	13	6	8	05/15/2014		View
309112		01/16/2014	05/22/2014	02/05/2014	17	7	8	4	8	03/24/2014		View
315717		01/30/2014	06/25/2014	03/21/2014	11	4	12	6	6	02/05/2014		View
316008		01/30/2014	06/16/2014	03/04/2014	15	8	16	6	7	03/26/2014		View
303116		02/04/2014	06/24/2014	06/24/2014	15	6	17	6	3	04/07/2014		View
311105		02/04/2014	07/07/2014	07/07/2014	18	14	12	18		07/07/2014		View
316312		02/05/2014	05/27/2014	04/23/2014	20	0	16	0	1	04/23/2014		View
318301		02/06/2014	06/03/2014	03/21/2014	16	4	12	3	2	02/19/2014		View
316705		02/10/2014	07/07/2014	07/07/2014	12	2	3	3	8	02/14/2014		View
321001		02/10/2014	03/19/2014	02/13/2014	5	6	14	8	1	02/13/2014		View
320608		02/11/2014	07/14/2014	07/14/2014	13	1	11	0	8			View
316505		02/14/2014	07/07/2014	05/07/2014	6	2	10	5	5			View
323606		02/18/2014	07/14/2014	07/14/2014	22	2	18	8	2	02/20/2014		View
321712		02/20/2014	04/28/2014	03/03/2014	9	3	10	6	1	03/03/2014		View
323304		02/21/2014	06/26/2014	05/08/2014	18	6	9	2	3	05/08/2014		View
325310		02/21/2014	07/07/2014	07/07/2014	13	6	12	1		03/03/2014		View
324913		02/24/2014	07/10/2014	07/10/2014	20	13	18	14	4	05/12/2014		View
325117		02/26/2014	07/11/2014	07/08/2014	15	3	5	5	7			View
325901		03/03/2014	07/10/2014	07/10/2014	3	1	17	4	1	03/17/2014		View
314217		03/04/2014	07/07/2014	07/07/2014	8	8	11	10				View
327412		03/04/2014	06/10/2014	04/01/2014	15	3	6	8	4	04/01/2014		View

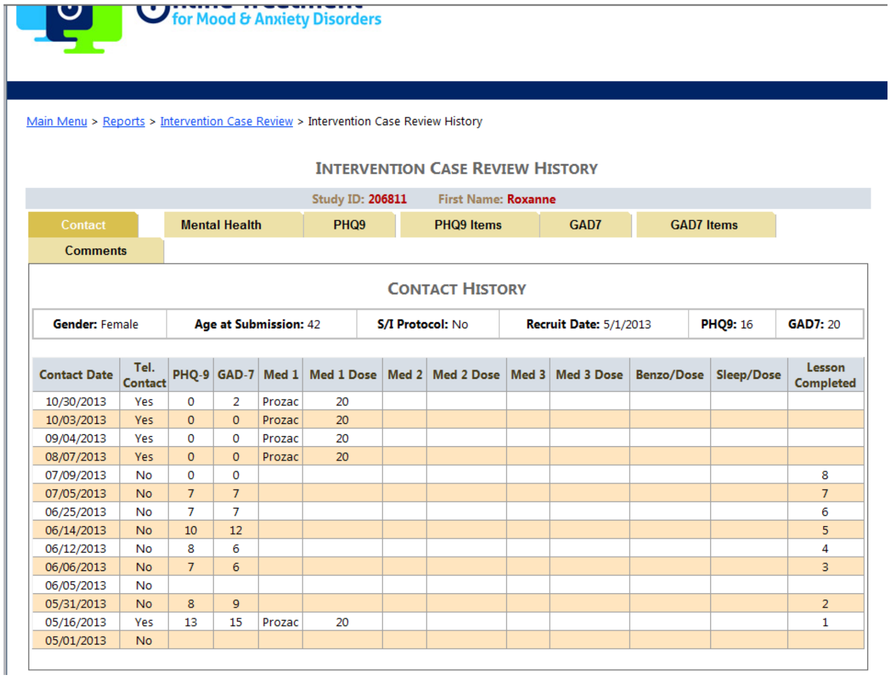

for Mood & Anxiety Disorders

Main Menu > Reports > Intervention Case Review > Intervention Case Review History

INTERVENTION CASE REVIEW HISTORY

Study ID: 206811 First Name: Roxanne

Contact | Mental Health | PHQ9 | PHQ9 Items | GAD7 | GAD7 Items | Comments

CONTACT HISTORY

Gender: Female	Age at Submission: 42	S/I Protocol: No	Recruit Date: 5/1/2013	PHQ9: 16	GAD7: 20

Contact Date	Tel. Contact	PHQ-9	GAD-7	Med 1	Med 1 Dose	Med 2	Med 2 Dose	Med 3	Med 3 Dose	Benzo/Dose	Sleep/Dose	Lesson Completed
10/30/2013	Yes	0	2	Prozac	20							
10/03/2013	Yes	0	0	Prozac	20							
09/04/2013	Yes	0	0	Prozac	20							
08/07/2013	Yes	0	0	Prozac	20							
07/09/2013	No	0	0									8
07/05/2013	No	7	7									7
06/25/2013	No	7	7									6
06/14/2013	No	10	12									5
06/12/2013	No	8	6									4
06/06/2013	No	7	6									3
06/05/2013	No											
05/31/2013	No	8	9									2
05/16/2013	Yes	13	15	Prozac	20							1
05/01/2013	No											

Online Treatment for Mood & Anxiety Disorders

Welcome **bea**! [Log Out] [Change Password]

Main Menu > Reports > Intervention Case Review > Intervention Case Review History

Intervention Case Review History

Study ID: 206811 First Name:

Contact | Mental Health | PHQ9 | PHQ9 Items | GAD7 | GAD7 Items | Comments

Mental Health History

**Past Anxiety:** No	**Past Depression:** Yes	**Alcohol:** No

History of mental health in family:

Anxiety-Who	Depression-Who	Bipolar/Manic-Who	Suicide (Attempts)-Who	Other-Who
Yes - mother	Yes - mother	No	No	No

Prior antidepressants and adverse reactions / side effects:

Name of Medication	Dose	Adverse or side effects
Sertaline (Zoloft)	25	very sedating
Paroxetine (Paxil)	10	made sx worse, compative too

Medical comorbidities: N/A
At risk for alcohol abuse: No
MH Referral: N/A

Screenshots B. CCBT program

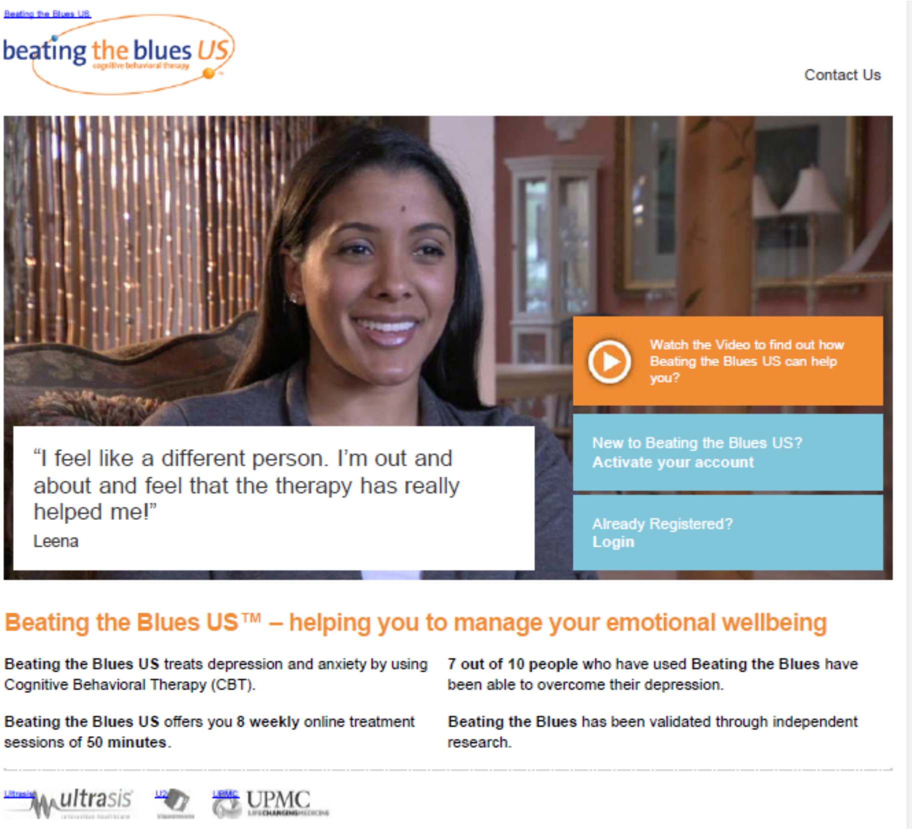

www.beatingthebluesus.com

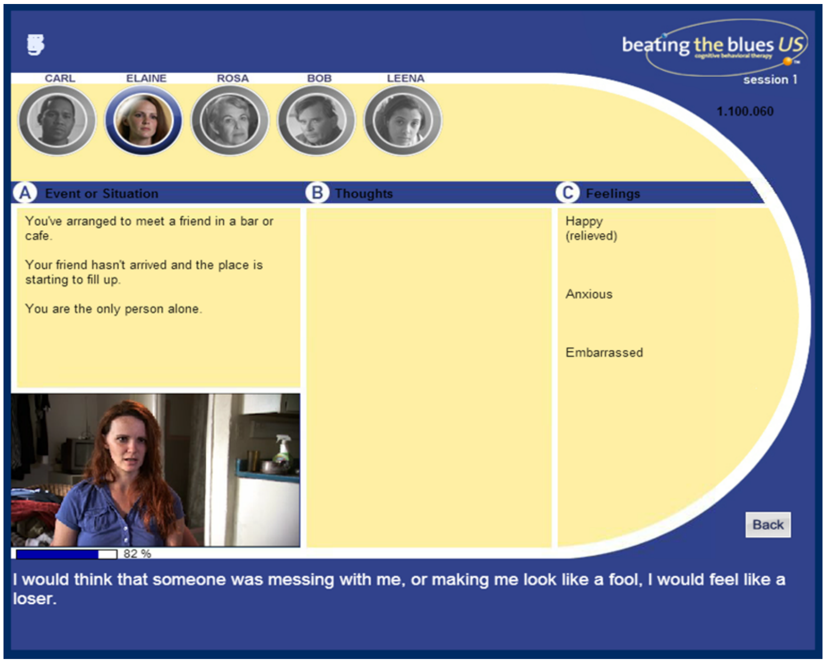
beating the blues US
session 1
CARL
ELAINE
ROSA
BOB
LEENA
1.100.060
A Event or Situation
B Thoughts
C Feelings
You've arranged to meet a friend in a bar or cafe.
Your friend hasn't arrived and the place is starting to fill up.
You are the only person alone.
Happy (relieved)
Anxious
Embarrassed
Back
82 %
I would think that someone was messing with me, or making me look like a fool, I would feel like a loser.

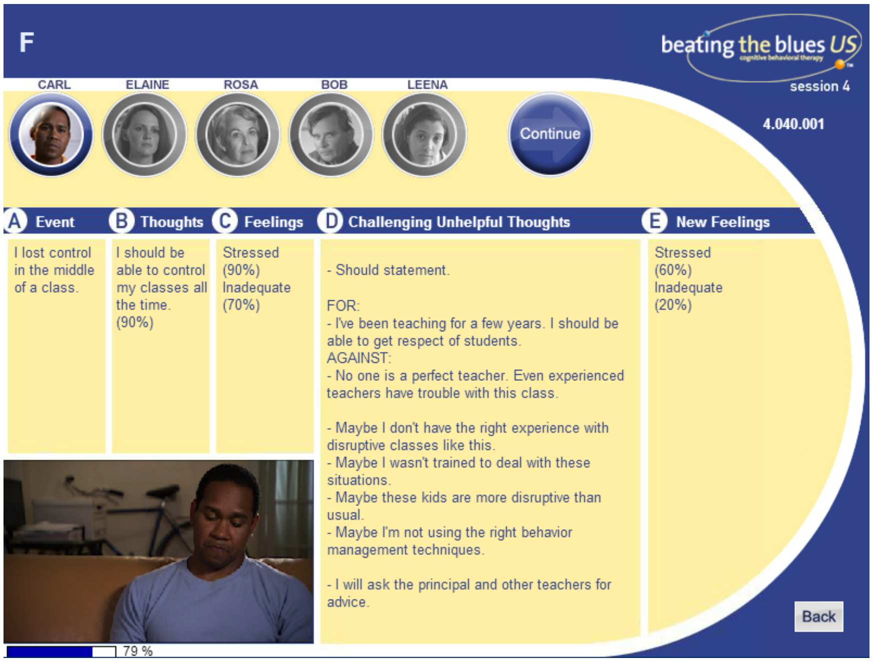
F
beating the blues US
session 4
CARL
ELAINE
ROSA
BOB
LEENA
Continue
4.040.001
A Event
B Thoughts
C Feelings
D Challenging Unhelpful Thoughts
E New Feelings
I lost control in the middle of a class.
I should be able to control my classes all the time. (90%)
Stressed (90%) Inadequate (70%)
- Should statement.
FOR:
- I've been teaching for a few years. I should be able to get respect of students.
AGAINST:
- No one is a perfect teacher. Even experienced teachers have trouble with this class.
- Maybe I don't have the right experience with disruptive classes like this.
- Maybe I wasn't trained to deal with these situations.
- Maybe these kids are more disruptive than usual.
- Maybe I'm not using the right behavior management techniques.
- I will ask the principal and other teachers for advice.
Stressed (60%) Inadequate (20%)
Back
79 %

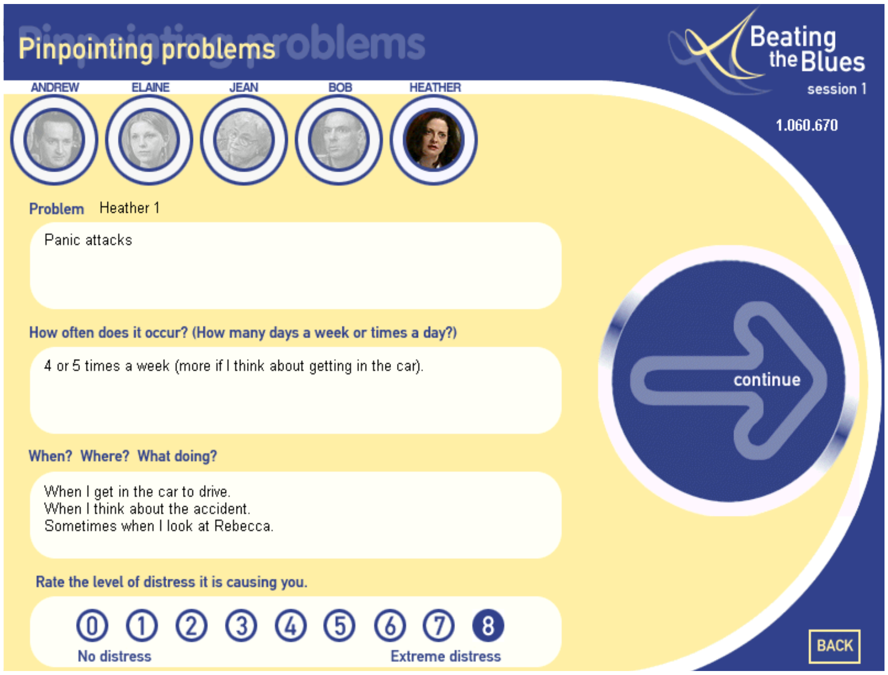
Pinpointing problems
Beating the Blues
session 1
1.060.670
ANDREW
ELAINE
JEAN
BOB
HEATHER
Problem Heather 1
Panic attacks
How often does it occur? (How many days a week or times a day?)
4 or 5 times a week (more if I think about getting in the car).
When? Where? What doing?
When I get in the car to drive.
When I think about the accident.
Sometimes when I look at Rebecca.
Rate the level of distress it is causing you.
0 1 2 3 4 5 6 7 8
No distress
Extreme distress
continue
BACK

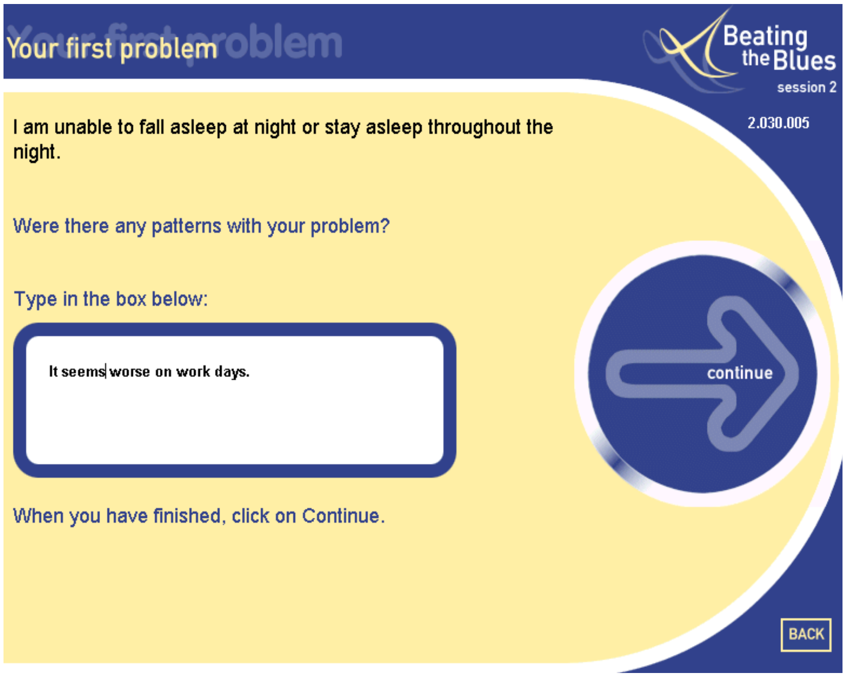
Your first problem
Beating the Blues
session 2
2.030.005
I am unable to fall asleep at night or stay asleep throughout the night.
Were there any patterns with your problem?
Type in the box below:
It seems worse on work days.
When you have finished, click on Continue.
continue
BACK

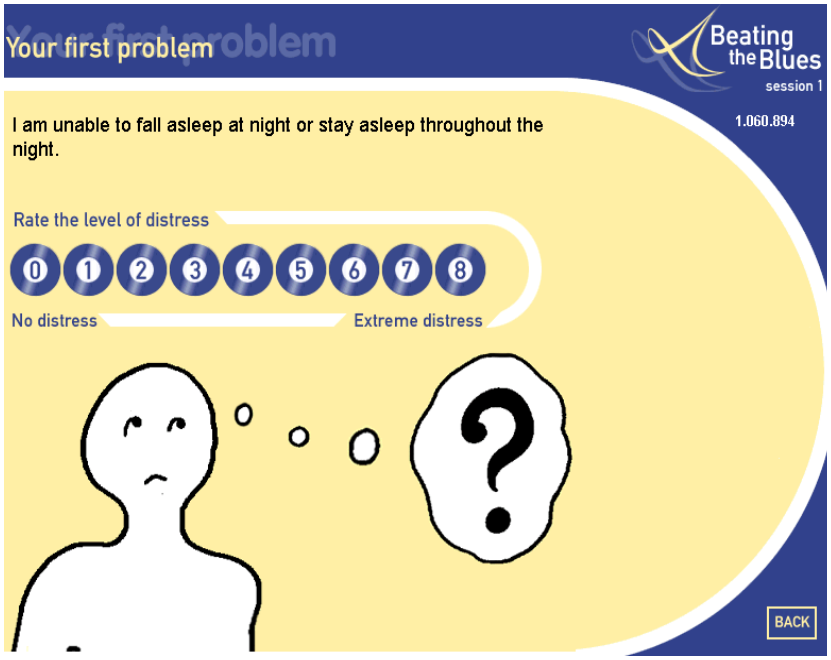
Your first problem
Beating the Blues
session 1
1.060.894
I am unable to fall asleep at night or stay asleep throughout the night.
Rate the level of distress
0 1 2 3 4 5 6 7 8
No distress
Extreme distress
BACK

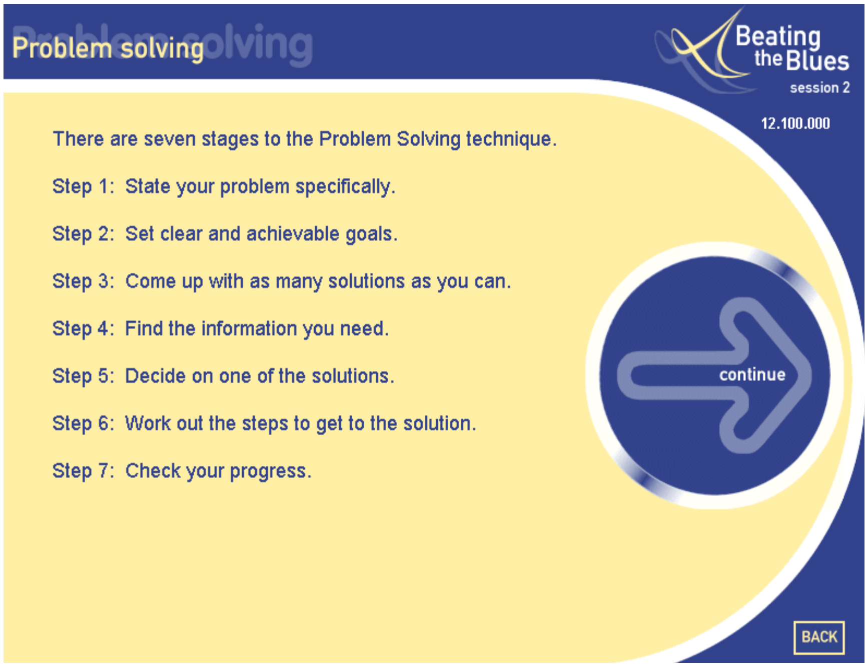
Problem solving
Beating the Blues
session 2
12.100.000
There are seven stages to the Problem Solving technique.
Step 1: State your problem specifically.
Step 2: Set clear and achievable goals.
Step 3: Come up with as many solutions as you can.
Step 4: Find the information you need.
Step 5: Decide on one of the solutions.
Step 6: Work out the steps to get to the solution.
Step 7: Check your progress.
continue
BACK

Care Manager Report

Problems

**Problem 1:**	Always tired, never have energy or motivation to do anything.
**Problem 2:**	I think about my past relationship all the time and how much it hurts and I wonder if I will have a relationship ever again.
**Problem 3:**	I wonder where I am going in life, will I ever be happy or content, will I ever find what I was born to do.

Table - Line Chart

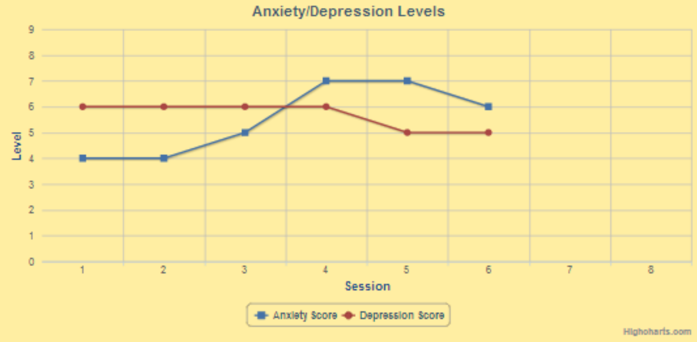

Table - Line Chart

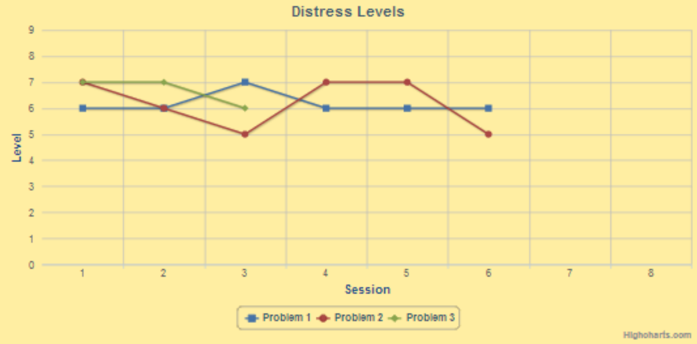

Screenshots C. Internet Support Group (ISG)

Homepage: Desktop version

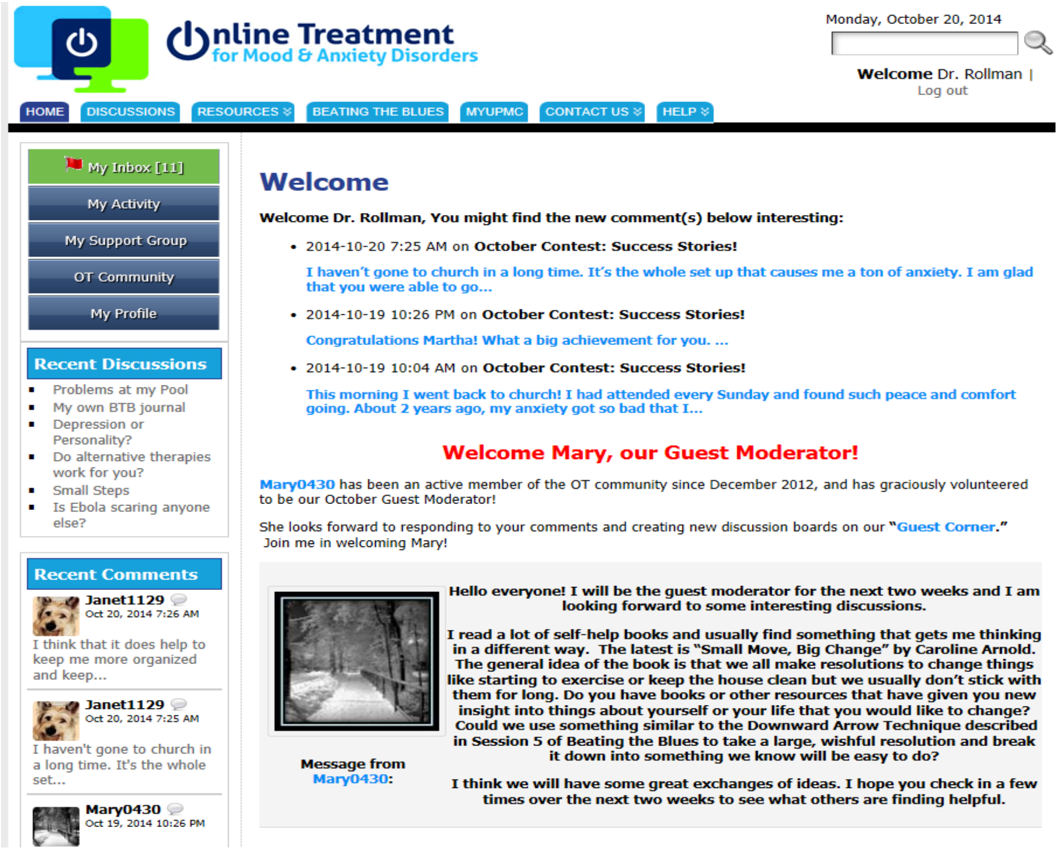

Homepage: Smartphone version

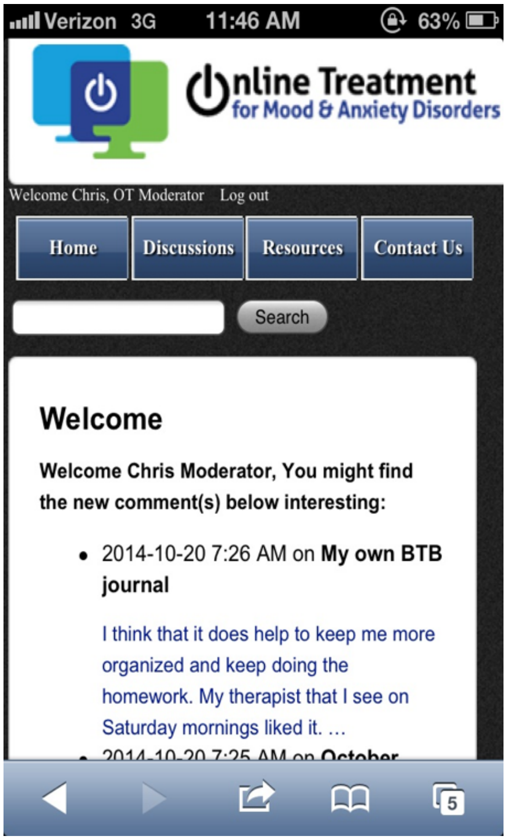

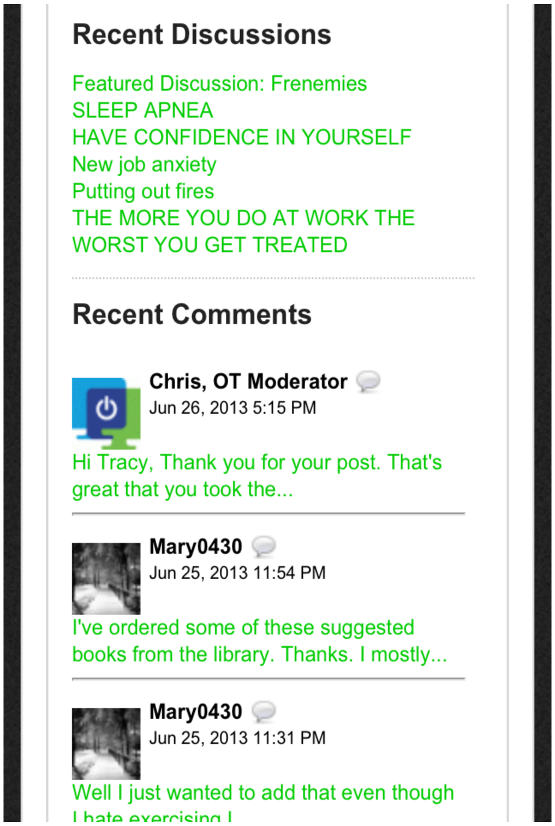

Discussion Boards

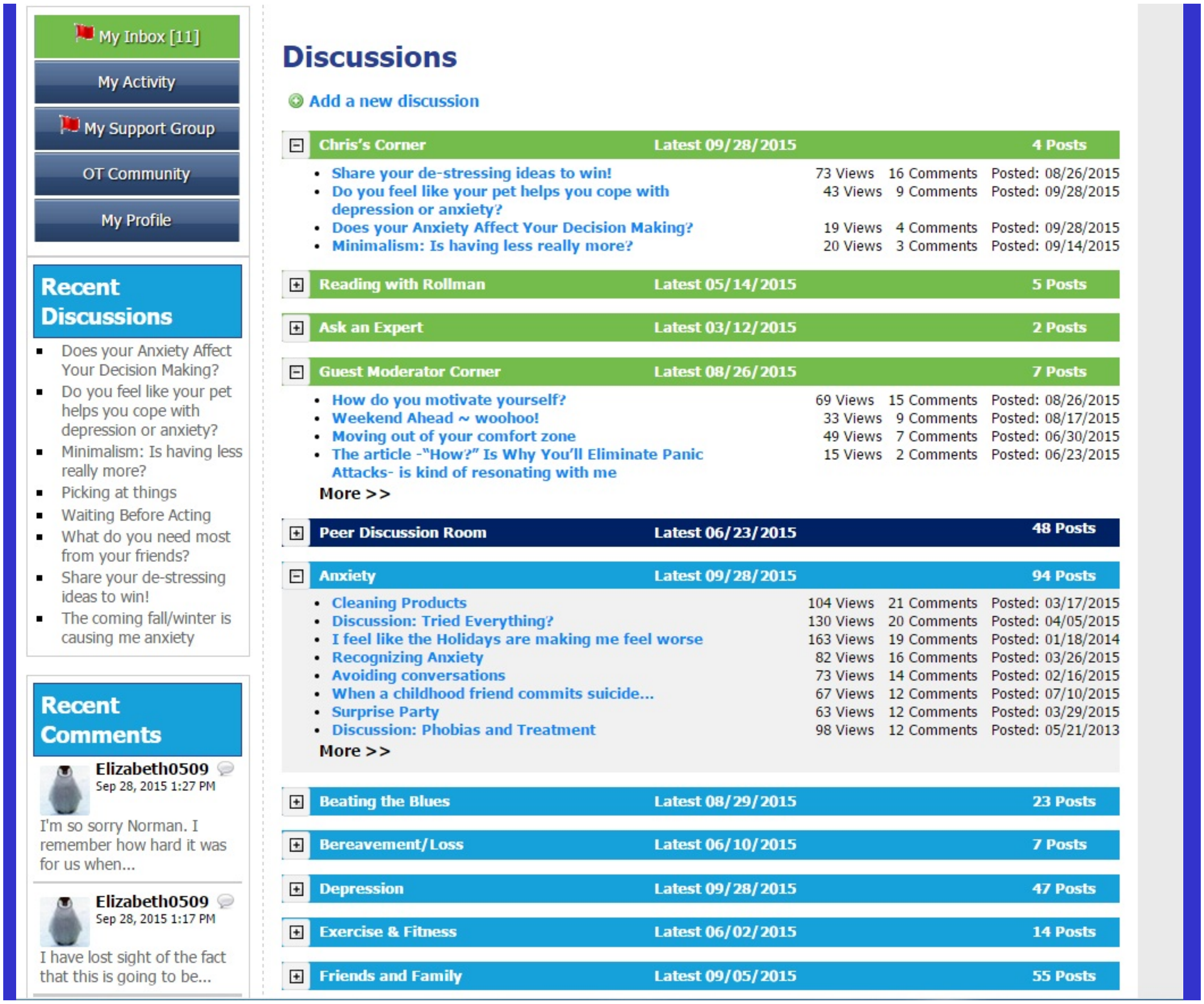

Resources

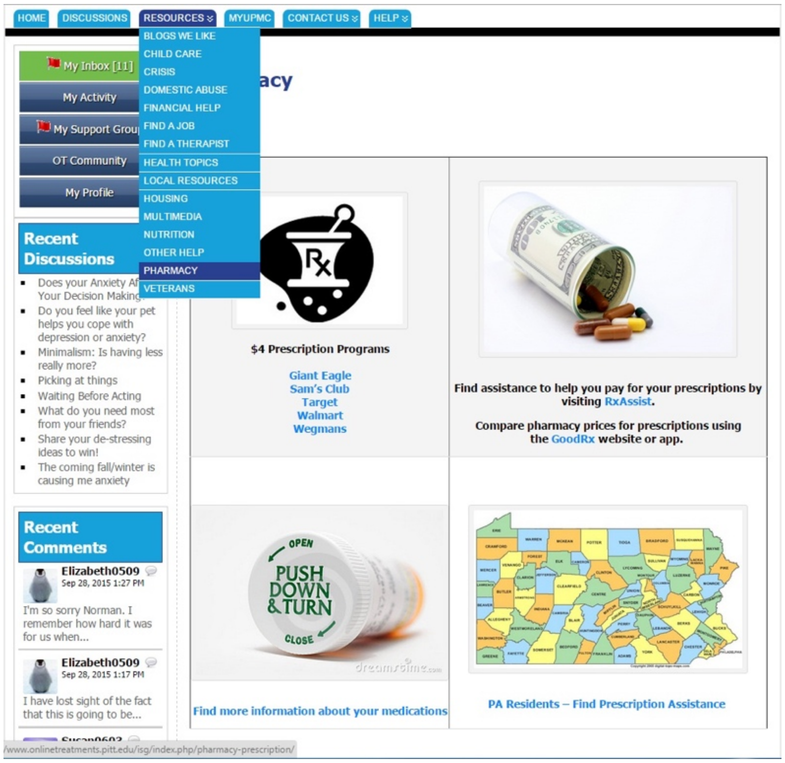
HOME
DISCUSSIONS
RESOURCES
MYUPMC
CONTACT US
HELP
BLOGS WE LIKE
CHILD CARE
CRISIS
DOMESTIC ABUSE
FINANCIAL HELP
FIND A JOB
FIND A THERAPIST
HEALTH TOPICS
LOCAL RESOURCES
HOUSING
MULTIMEDIA
NUTRITION
OTHER HELP
PHARMACY
VETERANS
My Inbox [11]
My Activity
My Support Grou
OT Community
My Profile
Recent Discussions
Does your Anxiety Af Your Decision Making
Do you feel like your pet helps you cope with depression or anxiety?
Minimalism: Is having less really more?
Picking at things
Waiting Before Acting
What do you need most from your friends?
Share your de-stressing ideas to win!
The coming fall/winter is causing me anxiety
Recent Comments
Elizabeth0509
Sep 28, 2015 1:27 PM
I'm so sorry Norman. I remember how hard it was for us when...
Elizabeth0509
Sep 28, 2015 1:17 PM
I have lost sight of the fact that this is going to be...
acy
$4 Prescription Programs
Giant Eagle
Sam's Club
Target
Walmart
Wegmans
Find assistance to help you pay for your prescriptions by visiting RxAssist.
Compare pharmacy prices for prescriptions using the GoodRx website or app.
OPEN
PUSH DOWN & TURN
CLOSE
Find more information about your medications
PA Residents – Find Prescription Assistance
/www.onlinetreatments.pitt.edu/isg/index.php/pharmacy-prescription/

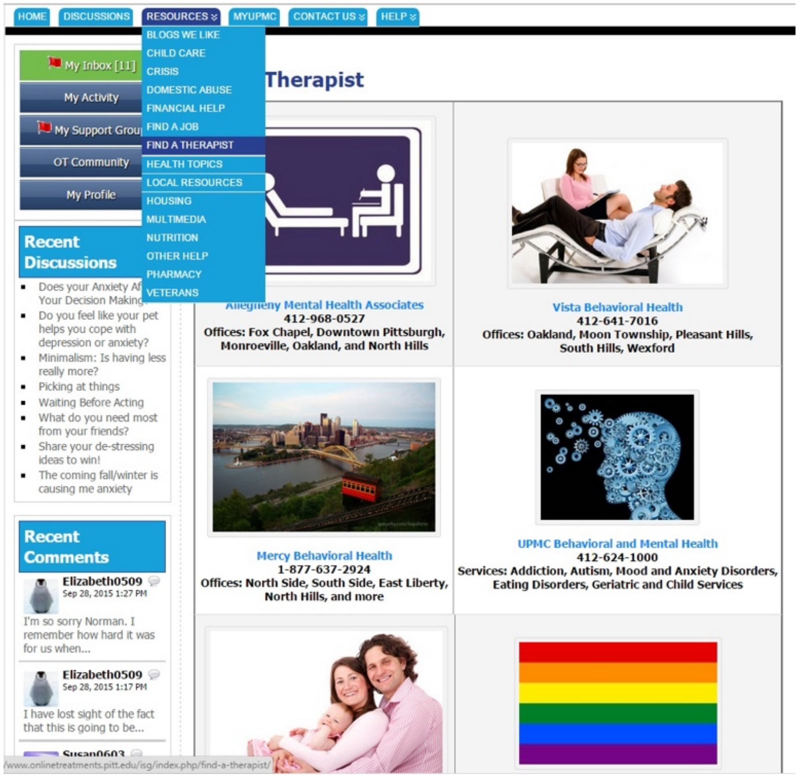
HOME
DISCUSSIONS
RESOURCES
MYUPMC
CONTACT US
HELP
BLOGS WE LIKE
CHILD CARE
CRISIS
DOMESTIC ABUSE
FINANCIAL HELP
FIND A JOB
FIND A THERAPIST
HEALTH TOPICS
LOCAL RESOURCES
HOUSING
MULTIMEDIA
NUTRITION
OTHER HELP
PHARMACY
VETERANS
My Inbox [11]
My Activity
My Support Grou
OT Community
My Profile
Recent Discussions
Does your Anxiety Af Your Decision Making
Do you feel like your pet helps you cope with depression or anxiety?
Minimalism: Is having less really more?
Picking at things
Waiting Before Acting
What do you need most from your friends?
Share your de-stressing ideas to win!
The coming fall/winter is causing me anxiety
Recent Comments
Elizabeth0509
Sep 28, 2015 1:27 PM
I'm so sorry Norman. I remember how hard it was for us when...
Elizabeth0509
Sep 28, 2015 1:17 PM
I have lost sight of the fact that this is going to be...
Therapist
Allegheny Mental Health Associates
412-968-0527
Offices: Fox Chapel, Downtown Pittsburgh, Monroeville, Oakland, and North Hills
Vista Behavioral Health
412-641-7016
Offices: Oakland, Moon Township, Pleasant Hills, South Hills, Wexford
Mercy Behavioral Health
1-877-637-2924
Offices: North Side, South Side, East Liberty, North Hills, and more
UPMC Behavioral and Mental Health
412-624-1000
Services: Addiction, Autism, Mood and Anxiety Disorders, Eating Disorders, Geriatric and Child Services
/www.onlinetreatments.pitt.edu/isg/index.php/find-a-therapist/

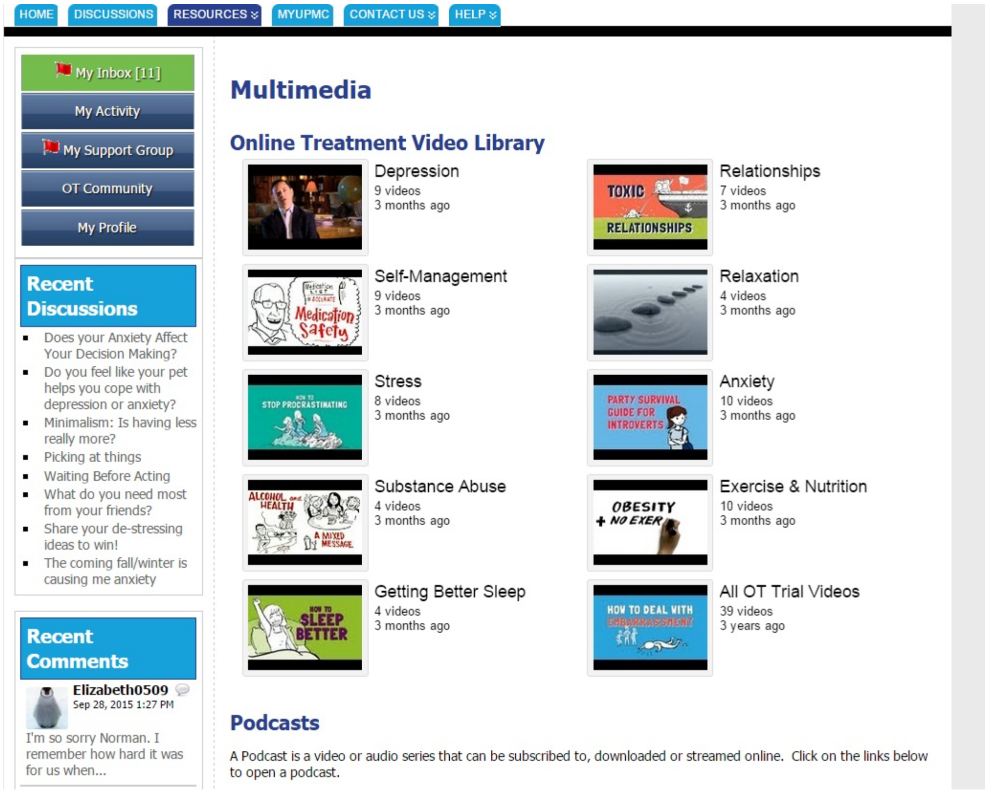

Patient Profile

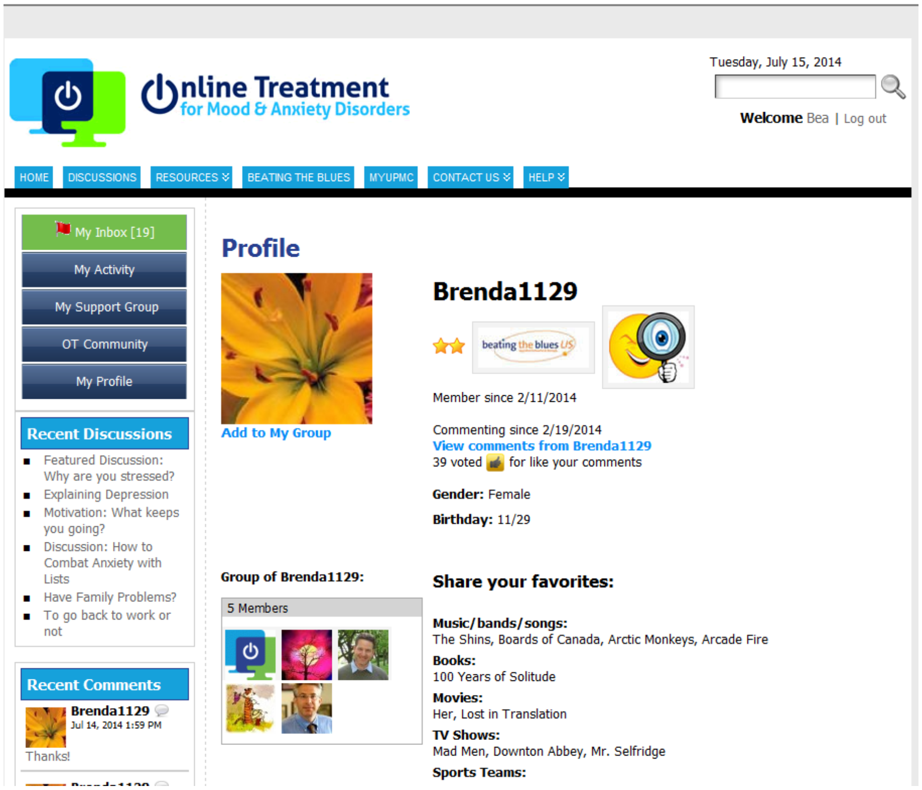

Supplement: Supplement 1. — Trial protocol. [file jamapsychiatry-75-56-s001.pdf]
