# Supplementary material for: Effectiveness of Online Collaborative Care for Treating Mood and Anxiety Disorders in Primary Care: A Randomized Clinical Trial
Source: JAMA Psychiatry. 2017 Nov 8;75(1):56–64. doi: 10.1001/jamapsychiatry.2017.3379 (PMC5833533; doi:10.1001/jamapsychiatry.2017.3379)
Supplement: Supplement 2. — eMethods. Statistical analysis plan (from funded grant application). eTable 1. Computerized cognitive behavior therapy sessions completed at 3 and 6 months following randomization. eTable 2. Effect size improvements by number of computerized cognitive behavior therapy (CCBT) sessions completed (CCBT alone vs usual care). eFigure 1. Screenshots of internet support group pages. eFigure 2. Boxplots of the number of logins, posts, comments, and posts or comments on internet support group. eFigure 3. Estimated scores by baseline treatment assignment for the SF-12 MCS by age. eFigure 4. Forest plots of between-group differences and effect sizes on the PROMIS Depression (top) and PROMIS Anxiety (bottom) scales. [file jamapsychiatry-75-56-s002.pdf]

Supplementary Online Content

Rollman BL, Herbeck Belnap B, Abebe KZ, et al. Effectiveness of online collaborative care for treating mood and anxiety disorders in primary care: a randomized clinical trial. *JAMA Psychiatry*. Published online November 8, 2017. doi:10.1001/jamapsychiatry.2017.3379

eMethods. Statistical analysis plan (from funded grant application)
eTable 1. Computerized cognitive behavior therapy sessions completed at 3 and 6 months following randomization
eTable 2. Effect size improvements by number of computerized cognitive behavior therapy (CCBT) sessions completed (CCBT alone vs usual care)
eFigure 1. Screenshots of internet support group pages
eFigure 2. Boxplots of the number of logins, posts, comments, and posts or comments on internet support group
eFigure 3. Estimated scores by baseline treatment assignment for the SF-12 MCS by age
eFigure 4. Forest plots of between-group differences and effect sizes on the PROMIS Depression (top) and PROMIS Anxiety (bottom) scales

This supplementary material has been provided by the authors to give readers additional information about their work.

© 2017 Rollman BL et al. *JAMA Psychiatry.*

eMethods. Statistical analysis plan (from funded grant application)

D14a. Sample Size and Power Analyses

Since we are targeting several disorders at once (depression, PD, and GAD), we require a measure that can assess a broad spectrum of function, health perceptions, and symptoms across conditions, and is responsive to alterations in mood and anxiety symptoms. Thus we selected the widely used and well-validated SF-12 Mental Component Summary (MCS) assessment of mental HRQoL as our primary outcome measure.[103]

Our primary hypothesis is that at 6-months follow-up, CCBT+ISG patients will report a clinically meaningful *0.30 ES* or greater intent-to-treat (ITT) improvement on the SF-12 MCS vs. CCBT-alone patients (Figure 1). Few data from trials of ISGs for mental health conditions are available to guide us.[10] Therefore, we selected this 0.30 ES estimate because of the: (1) 0.54 ES improvement in CES-D score described in Winzelberg et al.'s trial of an ISG for women with breast cancer,[108] the only controlled study identified in a 2009 review of ISGs for depression[10]; (2) 0.40 ES improvement in perceived stress observed in Dr. Rotondi's trial of an ISG for community-dwelling persons with schizophrenia[86, 109]; (3) 0.30 ES[2] and 0.38 ES[3, 4] improvements in SF-MCS scores we observed in our trials of collaborative care for post-CABG depression and anxiety, respectively; and (4) 0.30 ES is at the upper 95% CI of 6-month improvement in mood reported by a meta-analysis of collaborative care for treating depression in primary care (0.18-0.32) (B2).[6] If we assume a 90% 6-month assessment completion rate and a 2-tailed α=0.05, then if we enroll 300 subjects per arm (600 total patients) we will have 94% power to detect at least a 0.30 ES improvement of CCBT+ISG over CCBT-alone on the SF-12 MCS or any other continuous outcome measure (e.g., HRS-D).

To compare our on-line intervention to UC (secondary hypothesis), we estimate CCBT-alone subjects will report a moderate 0.50 ES or greater ITT improvement on the SF-12 MCS vs. "usual care". We base this on the: (1) 0.65 ES reported by Proudfoot et al.'s randomized trial of *Beating the Blues* versus "usual care"[53]; and (2) 0.50 ES (ITT) and 1.00 ES (program completers) in Cavanagh et al.'s naturalistic study of depressed and anxious patients.[62] We also note that a 0.50 ES improvement would compare favorably to non-CCBT trials of collaborative care described above. If we compare 300 intervention to 100 "usual care" subjects (Figure 1), then we will have ≥ 98% power to detect a 0.50 ES difference or greater at 6-months (2-tailed α=0.05; 90% 6-month assessment completion rate) and ≥ 80% power to detect a ≥ 0.34 ES difference on the SF-12 MCS.

D14b. Effectiveness of CCBT and ISG by Age

Data on how patients respond to CCBT and ISGs by age is limited[62] but of great interest. Thus, we will conduct exploratory analyses to evaluate the effectiveness of our interventions both within and across age strata. If we apply the age strata encountered in RELAX and assume 25% of our study cohort will be aged 60-75, then a 700 person total sample size (300 per on-line treatment arm) and 100 "usual care" subjects, will provide *within* the 60-75 age strata: (a) 82% power to detect an ES ≥ 0.50 of CCBT+ISG (n=75) vs. CCBT (n=75); (b) 80% power to detect an ES ≥ 0.70 of CCBT (N=75) vs. UC (n=25); and (c) 80% power to detect a Cohen's f ES ≥ 0.30 (using F-test) for treatment X age interactions using two treatment arms (CCBT and CCBT+ISG) and three age strata (18-34, 35-59, and 60-75).

D14c. African-American and Minority Patients

We expect to enroll 25% non-Caucasian patients of whom 80% will be African-American (20% overall) reflecting the demographics of the Pittsburgh region. If we randomize 60 African-American patients to each on-line intervention and 20 to UC, then we will have 80% power to detect an ES ≥ 0.54 difference between CCBT and CCBT+ISG, and 80% power to detect an ES ≥ 0.77 between CCBT and UC.

D15a. Data Analyses - Overview
We will use an ITT perspective to estimate the magnitude of benefits that can be expected in routine clinical practice with mixed-effects repeated measures models as our main analytic tool. Beforehand, we will examine our data by study arms in univariate fashion using cross tabulations, histograms, and box plots, and check for practice site differences applying standard statistical tests, and adjusting for baseline differences by randomization status where appropriate. We will investigate the reasons for intermittently missing data (misses an assessment but comes back) and dropouts, and use a likelihood-based or multiple imputation-based procedure if "missing at random" (MAR) is confirmed[110] or consider selection models with a logistic dropout process if the missingness is found to be nonignorable (missing not at random (MNAR)).[111] Although we will not use such ITT methods as last-observation-carried-forward (LOCF), we will conduct an adjusted completer analysis using an instrumental variable approach to reduce the effects of confounding,[112] with sensitivity analyses to examine the robustness of our findings.

D15b. Clinical Effectiveness, Moderators, Mediators, Adoption, and Maintenance
We will use ITT mixed-effects repeated measures models with time (random) and treatment to evaluate the impact of our interventions both to each other and CCBT to UC (Figure 1). To model our data appropriately, we will also include several potential moderators of treatment response measured prior to randomization that may either potentiate or attenuate the effects of our interventions (e.g., patient gender, age strata, race, baseline symptom severity, medical co-morbidity; PCP knowledge and attitudes) in a series of 3-way interactions that include time, treatment, and the moderator of interest[113] to identify patient and provider subgroups for whom our interventions may be particularly effective.

To better understand how patients use the elements of our interventions and which may be most effective, we will conduct exploratory *post-hoc* analyses of several measures of "dose" as a mediator of treatment effects (e.g., number of CCBT sessions completed; ISG log-ins, posts, and page views; number of care manager contacts; use of pharmacotherapy; PCP visits; MHS referral). We will use Kraemer's methodology to assess if one or more of these factors is intermediate in a casual relationship between an independent variable and dependent variable and therefore a mediator of our observed outcomes by evaluating whether the "dose": (1) is related to treatment; (2) is associated with the outcome; and (3) improves prediction of the outcome after adjustment for treatment arm.[113]
In keeping with PRISM (D12), we will also evaluate the adoption and maintenance of our interventions following the Intervention Phase of the Project (D18) and attempt to link them to information collected on various organizational facilitators and barriers (E2a). To do so, we will retain the EpicCare BPA (D4) during the Post-Intervention Phase and direct referred patients to CCBT+ISG (Table 1), and then analyze usage statistics (e.g., BPA referrals, ISG log-ins) and changes in PHQ-9 and GAD-7 scores that are automatically captured by the EMR and CCBT programs (D8b).

© 2017 Rollman BL et al. *JAMA Psychiatry.*

Final Statistical Analysis Plan

Study Design & Objectives. This a 4-year comparative effectiveness trial that will randomize 700 primary care patients aged 18-75 who have at least a moderate level of mood and/or anxiety symptoms and reliable access to both the Internet and e-mail to either: (1) guided patient access to *Beating the Blues*, a proven-effective on-line CCBT program (CCBT-alone; N=300); (2) guided patient access to *Beating the Blues* plus access to a moderated ISG (CCBT+ISG; N=300); or (3) their PCP's "usual care" (N=100). Our primary hypothesis is that patients in our CCBT+ISG arm will report a clinically meaningful 0.30 effect size (ES) or greater improvement in HRQoL on the SF-12 MCS compared to patients in our CCBT-alone arm at 6-months follow-up, and we will monitor patients for an additional 6 months to evaluate the durability of our interventions. Our secondary hypothesis is that CCBT-alone patients will report a 0.50 ES or greater improvement in HRQoL on the SF-12 MCS versus "usual care" at 6-months follow-up To better understand how online mental health treatments are best provided through primary care, we will also evaluate: (a) their effectiveness across and within age strata; (b) their cost-effectiveness; (c) how patients utilize the components of our interventions; (d) patient subgroups for whom our interventions may be particularly effective; and (e) the adoption and maintenance of our interventions by practices following the Intervention Phase of the Project. Study findings are likely to have profound implications for transforming the way mental health conditions are treated in primary care and focus further attention to the emerging field of e-mental health by other U.S. investigators.

Sample Size Calculation. Power for this trial will be based on the primary hypothesis that CCBT+ISG patients will report a clinically meaningful 0.30 6-month effect size (Cohen's d ES) improvement on the SF-12 mental component score (MCS), our primary outcome measure, versus CCBT-alone. We powered the CCBT+ISG vs CCBT-alone comparison of our trial to detect a 0.30 ES difference based on meta-analyses of the effectiveness of collaborative care interventions (manuscript ref #1, Archer J. et al. *Cochrane Database Syst Rev. 2012; 10:CD006525*), and our prior collaborative care trials (manuscript refs #30-32). Assuming a 90% 6-month assessment completion rate and 2-tailed type I error of 0.05, randomizing 300 subjects per arm (600 total) would provide 90% power to detect a 0.30 ES improvement of CCBT+ISG over CCBT-alone (primary hypothesis) and 0.50 ES improvement of CCBT-alone over UC (secondary hypothesis) on the SF-12 MCS or our other continuous outcome measures (e.g., PROMIS Mood). We powered the CCBT-alone vs. UC comparison of our trial to detect a 0.50 ES difference based on meta-analyses of the effectiveness of other CCBT programs for treating mood and anxiety disorders (manuscript refs #3-4).

As patient engagement and response to our interventions could differ by age, we plan to test our primary hypothesis within three age strata: 18-34, 35-59, and 60-75. Assuming our smallest age strata is 25% of our study cohort (60-75) and our other assumptions, then randomizing 150 patients would provide 80% power to detect an ITT ES ≥ 0.50 on our continuous measures within our smallest age strata. Applying these assumptions to our secondary hypotheses (CCBT-alone versus UC), we need to randomize 100 UC patients to have 80% power to detect a 0.70 ES improvement on the SF-12 MCS within our smallest age strata.

Interim & Final Analyses. This study will not have planned interim looks for the primary outcome. The final analyses will be conducted once study follow-up is complete, after all data is cleaned, and once the study database is locked.

Hypotheses. Our primary hypothesis is that at 6-months follow-up, CCBT+ISG patients will report a clinically meaningful 0.30 effect size (ES) or greater improvement on the SF-12 MCS versus CCBT-alone patients. To compare CCBT-Alone to Usual Care (secondary hypothesis), we anticipate that CCBT-alone subjects will report a moderate 0.50 ES or greater ITT improvement on the SF-12 MCS vs. Usual Care.

© 2017 Rollman BL et al. *JAMA Psychiatry.*

Analysis Sets. The full analysis set will be based on an intention-to-treat (ITT) analysis, which will comprise all participants who have been randomized to any of the 3 study arms, regardless of length of follow-up or actual intervention received. As an exploratory measure, we will conduct per-protocol (PP) analyses for the secondary hypothesis using various definitions based on the number of CCBT sessions. These PP analysis sets will be based on those definitions (i.e. including participants with all 8 sessions completed).

Study Outcomes. The primary outcome is mental health-related quality of life (HRQoL) as defined by the SF-12 Mental Component Score (MCS). Secondary outcomes include mood and anxiety, as measured by the PROMIS Depression and Anxiety scales.

Handling of Missing Values. As a preventive measure, we will make every attempt to document all reasons for missing data. In addition, baseline characteristics will be compared between participants who do and do not withdraw from the study as a way to assess the impact of missing information and attrition. We will also compare the rates of lost-to-follow-up (LTF) between study arms.

We will investigate the reasons for intermittently missing data (misses an assessment but comes back) and dropouts, and use a likelihood-based or multiple imputation-based procedure if "missing at random" (MAR) is confirmed[110] or consider selection models with a logistic dropout process if the missingness is found to be nonignorable (missing not at random (MNAR)).[111]

Statistical Analyses. Demographic and baseline characteristics will be presented as mean and standard deviations for continuous variables and sample proportions for categorical variables. All descriptive statistics will be accompanied by 95% confidence intervals. Baseline comparisons between study arms (for each hypothesis) will be performed using t-tests for continuous variables, chi-squared tests for categorical variables, or their nonparametric counterparts. Diagnostics (i.e. conditional studentized residuals, plots of outcomes over time) were performed on each linear mixed model to assess whether necessary assumptions were met.

The primary analyses will consider an intention-to-treat (ITT) analysis with exploratory per-protocol (PP) analyses to supplement the main findings. The ITT and PP analysis sets have been defined previously.

<u>Primary Outcome</u>: The ITT analyses will assess the effect of CCBT+ISG (versus CCBT alone) and CCBT alone (versus Usual Care) on 6-month improvements in mental health-related quality of life (HRQoL), as measured by the SF-12 MCS. We will fit a Laird and Ware linear mixed model [1] as a function of the following predictors: study arm, time, time-by-study arm, age group (18-34, 35-64, and 65+), and clinic size (small or large as defined by a size of 6 PCPs). In addition, the intercept will be allowed to vary randomly to account for subject-level variability of the outcome at baseline (We considered a random coefficients model with random intercept and slope and independent covariance structure, but convergence issues led us to use linear mixed models with a random intercept terms instead.). The primary hypothesis will involve contrasts to estimate the adjusted mean difference in 6-month improvement on SF-12 MCS between CCBT+ISG and CCBT alone study arms. The secondary hypothesis will be similar, but between CCBT alone and Usual Care study arms.

<u>Secondary Outcomes</u>: Similar to SF-12 MCS, a Laird and Ware linear mixed model will be fit to estimate the adjusted mean differences in 6-month improvement on PROMIS-Depression and PROMIS-Anxiety.

<u>Subgroup and Exploratory Analyses</u>: Planned subgroup analyses will be conducted for the primary outcome of SF-12 MCS as well as secondary outcomes. A formal hypothesis will be tested with an interaction between each of the study arms and the following subgroups: 1) age group (18-34, 35-59, and 60-75), 2) gender, 3) race (white versus non-white), 4) baseline symptom severity (GAD-7 <15 vs >=15 and PHQ-9 <15 versus >=15), and 5) clinic size (small versus large). A significant 3-way

interaction between time, study arm, and the potential covariate will indicate a subgroup effect.
In order to investigate a potential “dose-response” in the secondary hypothesis (CCBT-Alone versus Usual Care), we will assess the effect of the number of completed BtB sessions has on the primary and secondary outcomes. We will use the same linear mixed model mentioned above, but will parametrize the study arm based on the number of sessions completed. For example, instead of 1 and 0 for CCBT-Alone and Usual Care, respectively, participants in the CCBT-Alone arm will receive a value equal to the proportion of 8 sessions completed. Those who completed none as well as those in the Usual Care arm will receive a value of 0.

Major Revisions to the SAP

1. One of the stratification variables for randomization was based on age group with the intended grouping being: 18-34, 35-59, and 60-75 years of age. As a result of an error unknown to study team until Dec 2016, the actual stratification categories were: 18-34, 35-64, and 65+. All primary and secondary analyses were analyzed as designed with the latter age category used as a covariate in the linear mixed models (along with site size). Only for Table 1 and the age subgroup analysis did we use the originally intended age groupings solely for presenting meaningful age groups.
2. Addition of key secondary outcomes in PROMIS Depression and Anxiety.
3. Reformatting of SAP layout.

References:

1. Laird NM, Ware JH. Random-effects models for longitudinal data. *Biometrics*. 1982;38:963-974. doi:10.2307/2529876.

© 2017 Rollman BL et al. *JAMA Psychiatry.*

eTable 1. Computerized cognitive behavior therapy sessions completed at 3 and 6 months following randomization

	**At 3 Months*<br>Mean (SD)<br>Completed 1+ sessions<br>% (N)**	**At 6 Months**<br>Mean (SD)<br>Completed 1+ sessions<br>% (N)**
Gender (N)		
Male (124)	4.7 (2.6)<br>80% (99)	5.4 (2.7)<br>81% (101)
Female (479)	4.7 (2.6)<br>83% (396)	5.4 (2.7)<br>84% (403)
Race (N)		
White (499)	4.8 (2.5)<br>84% (421)	5.5 (2.7)<br>86% (429)
Non-White (104)	4.4 (2.7)<br>71% (74)	5.0 (2.7)<br>72% (75)
Age, years (N)		
18-34 (219)	4.6 (2.6)<br>81% (177)	5.3 (2.8)<br>82% (179)
35-59 (292)	4.7 (2.5)<br>82% (238)	5.5 (2.6)<br>84% (244)
60-75 (92)	5.2 (2.8)<br>87% (80)	5.6 (2.9)<br>88% (81)

* Of those who completed ≥ 1 session within the first 3 months following randomization.

** Of those who completed ≥ 1 session within the 6-month intervention period.

© 2017 Rollman BL et al. *JAMA Psychiatry.*

eTable 2. Effect size improvements by number of computerized cognitive behavior therapy (CCBT) sessions completed (CCBT alone vs usual care)

	Intent-to-Treat† ES (95% CI) N=301	≥4 CCBT Sessions Completed ES (95% CI) N=176	All 8 CCBT Sessions Completed ES (95% CI) N=112
SF-12 MCS	0.15 (-0.06 - 0.36)	0.27 (0.05 - 0.49)*	0.42 (0.19 - 0.66)**
PROMIS Depression	0.31 (0.09 - 0.53)*	0.41 (0.17 - 0.65)**	0.52 (0.26 - 0.78)**
PROMIS Anxiety	0.26 (0.05 - 0.48)*	0.34 (0.10 - 0.57)*	0.49 (0.24 - 0.75)**

† - vs. UC, Mixed model, N=402;

* P<0.02,

** P<0.001

Abbreviations: CCBT, computerized cognitive behavioral therapy; PROMIS Anxiety, Patient-Reported Outcomes Measurement Information System for Anxiety (fixed length, short form); PROMIS Depression, Patient-Reported Outcomes Measurement Information System for Depression (fixed length, short form); SF-12 MCS, Medical Outcomes Study Short Form Mental Health Composite Scale;

© 2017 Rollman BL et al. *JAMA Psychiatry.*

eFigure 1. Screenshots of internet support group pages

Desktop version

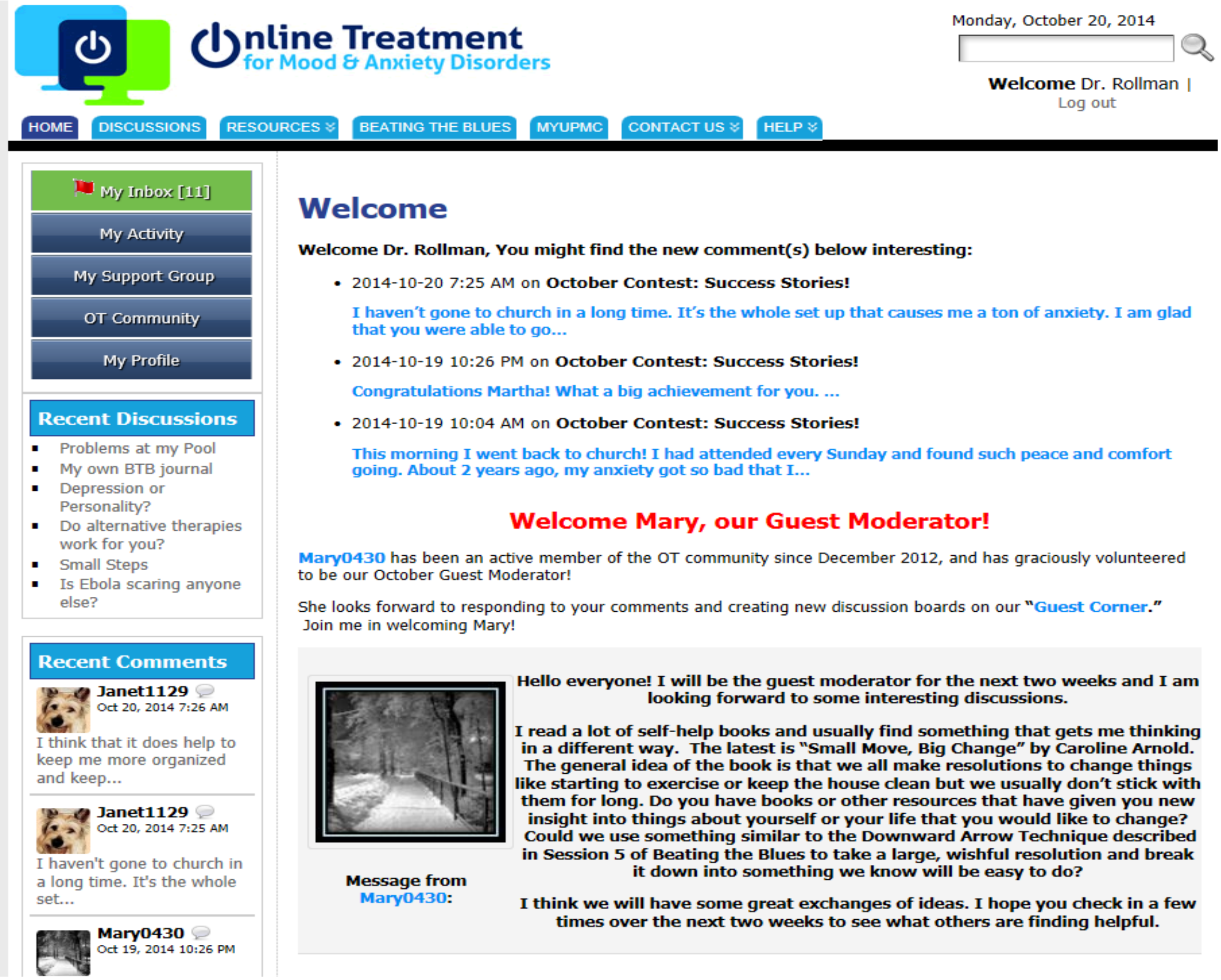

© 2017 Rollman BL et al. *JAMA Psychiatry.*

Smartphone version

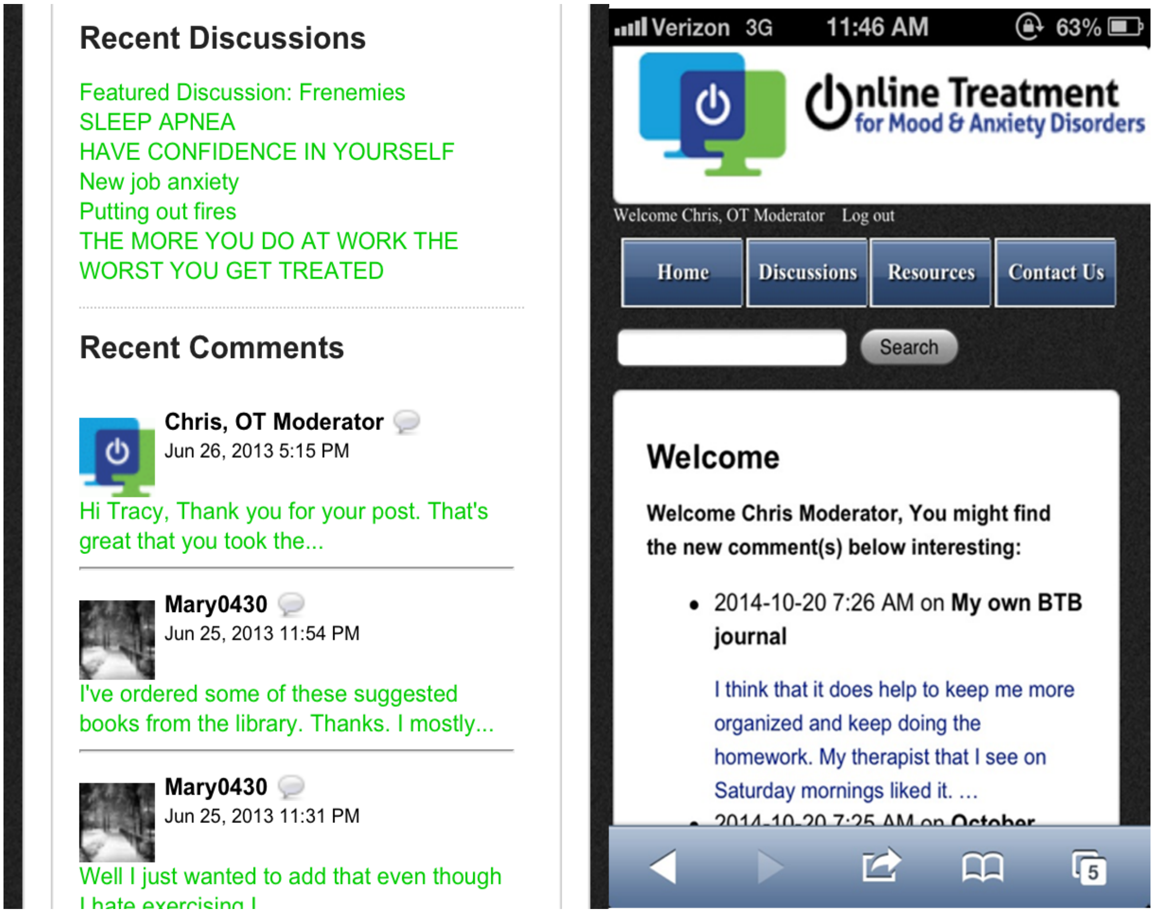

© 2017 Rollman BL et al. *JAMA Psychiatry.*

Discussion Boards

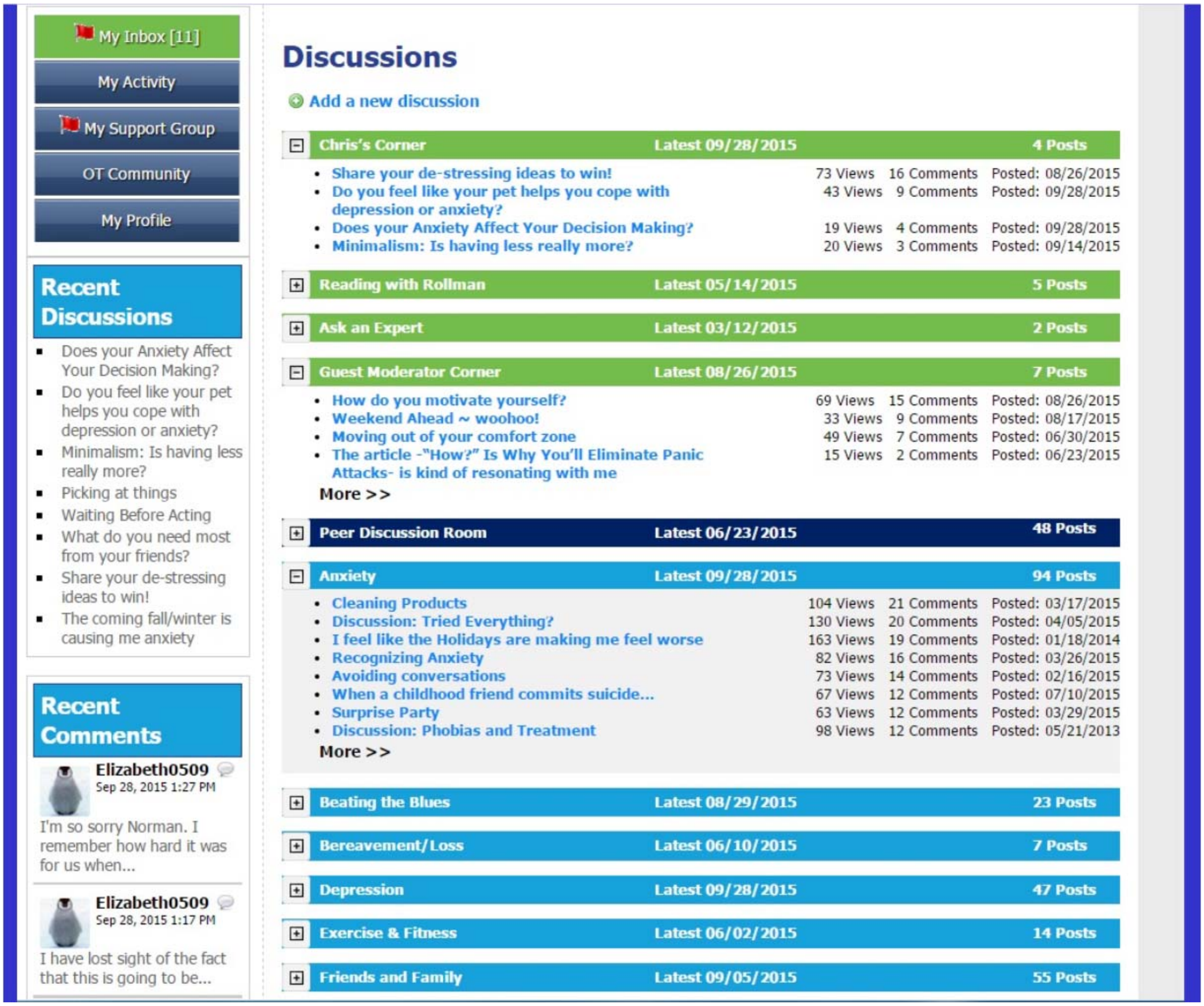

© 2017 Rollman BL et al. *JAMA Psychiatry.*

Resources

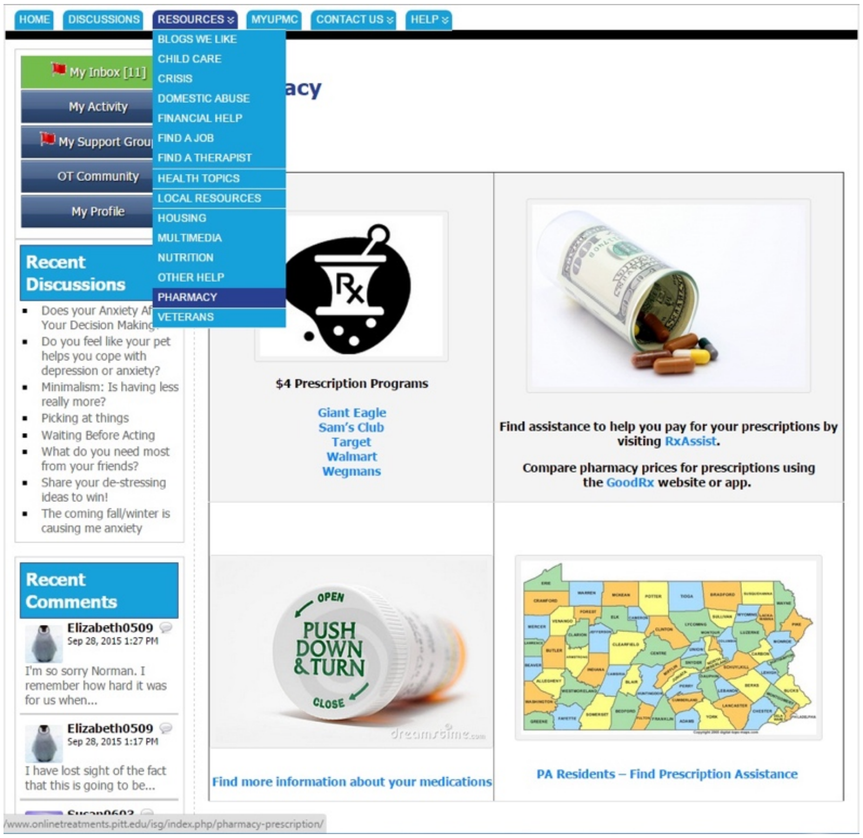

© 2017 Rollman BL et al. *JAMA Psychiatry.*

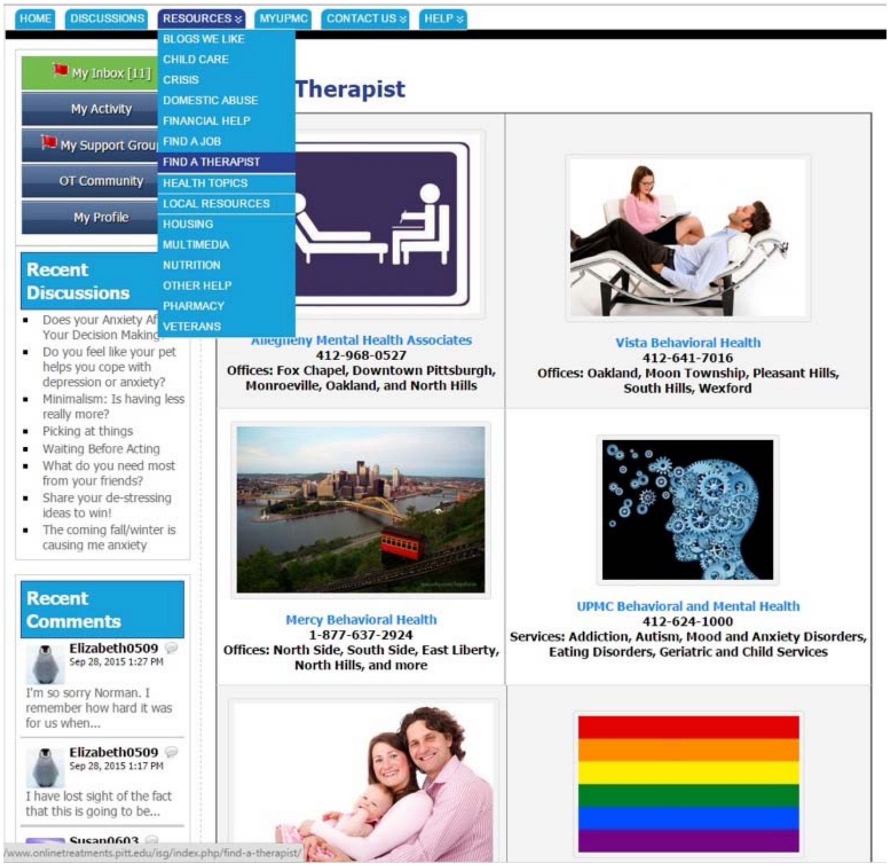

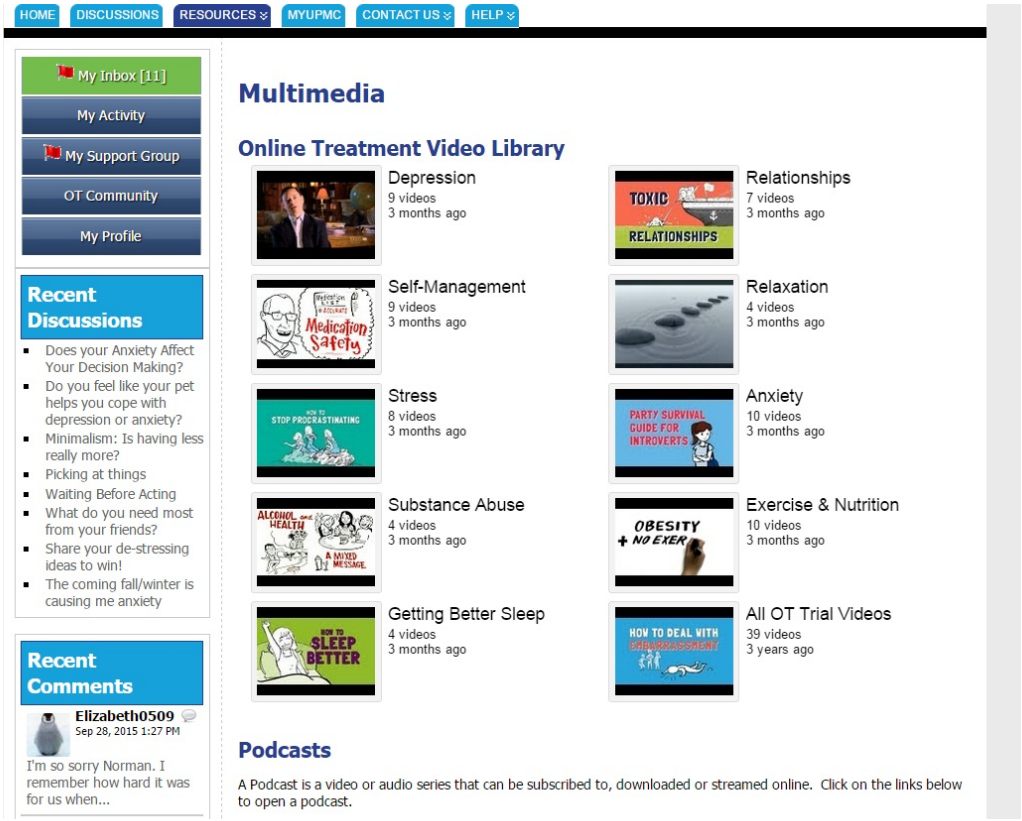

© 2017 Rollman BL et al. *JAMA Psychiatry.*

Patient Profile

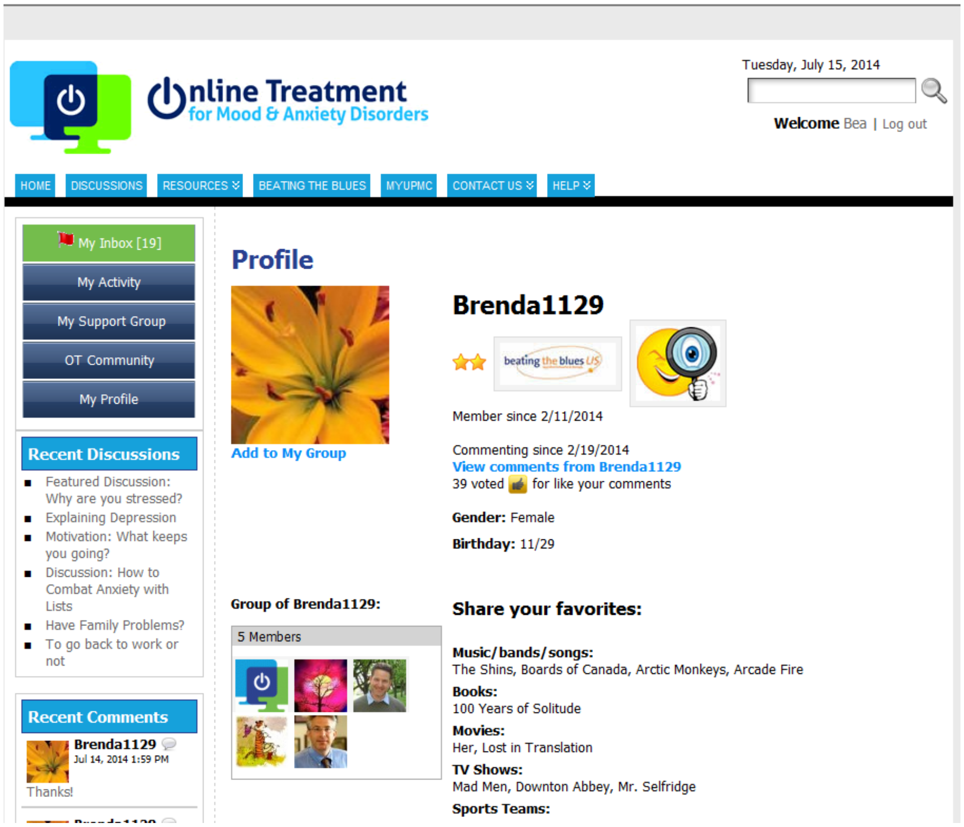

© 2017 Rollman BL et al. *JAMA Psychiatry.*

eFigure 2. Boxplots of the number of logins, posts, comments, and posts or comments on internet support group

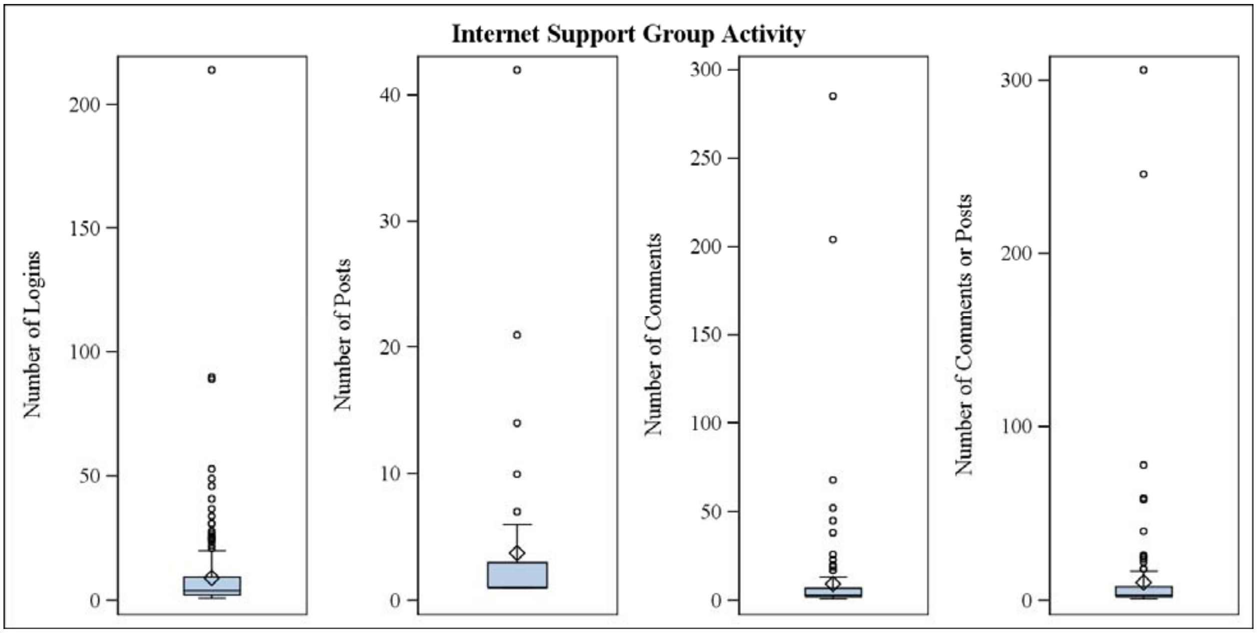

© 2017 Rollman BL et al. *JAMA Psychiatry.*

eFigure 3. Estimated scores by baseline treatment assignment for the SF-12 MCS by age

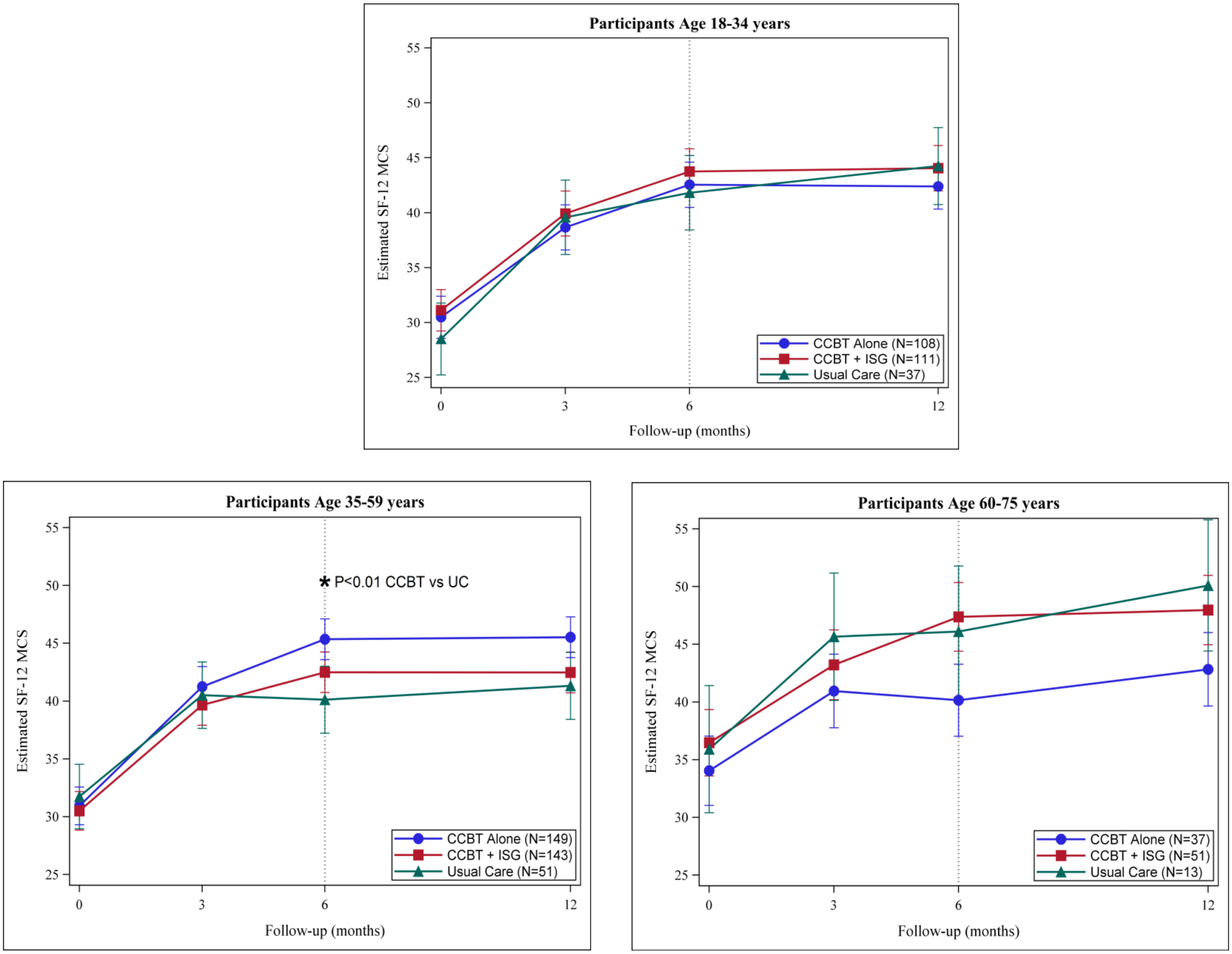

Linear mixed models adjusted for time, study arm, time-by-study arm, age strata, and clinic size. The vertical line at 6 months indicates the end of care manager-led CCBT and our primary outcome time point. The following 6-months were naturalistic follow-up.

© 2017 Rollman BL et al. *JAMA Psychiatry.*

eFigure 4. Forest plots of between-group differences and effect sizes on the PROMIS Depression (top) and PROMIS Anxiety (bottom) scales

Subgroup	# Patients (%) CCBT+ISG	CCBT	UC	Between Group Diff (95%CI) CCBT+ISG vs CCBT	P-Value	Between Group Diff (95%CI) CCBT vs UC	P-Value
Overall	302	301	101	0.56 (-0.69, 1.81)	0.378	-2.43 (-4.16, -0.69)	0.006
Age					0.002		0.033
18 - 34 yr	111 (37)	108 (36)	37 (37)	-0.67 (-2.76, 1.42)		-0.04 (-2.88, 2.81)	
35 - 59 yr	143 (47)	149 (50)	51 (50)	2.34 (0.56, 4.12)		-4.25 (-6.68, -1.82)	
60 - 75 yr	48 (16)	44 (15)	13 (13)	-2.20 (-5.30, 0.90)		-2.63 (-7.29, 2.02)	
Race					0.191		0.024
White	242 (80)	257 (85)	77 (76)	0.38 (-1, 1.76)		-1.73 (-3.71, 0.24)	
Non-White	60 (20)	44 (15)	24 (24)	1.5 (-1.45, 4.46)		-4.79 (-8.47, -1.10)	
Gender					0.422		0.803
Male	57 (19)	66 (22)	19 (19)	-1.01 (-3.76, 1.74)		-2.22 (-6.23, 1.79)	
Female	245 (81)	235 (78)	82 (81)	0.96 (-0.44, 2.36)		-2.51 (-4.43, -0.59)	
Education					0.259		0.062
< 4 yr college degree	158 (52)	164 (54)	49 (49)	1.19 (-0.52, 2.9)		-4.39 (-6.82, -1.97)	
>= 4 yr college degree	144 (48)	137 (46)	52 (51)	-0.19 (-2.02, 1.63)		-0.32 (-2.79, 2.14)	
Practice Size					0.888		0.829
<= 6 PCPs	116 (38)	116 (39)	39 (39)	-0.01 (-2.02, 2)		-3.34 (-6.11, -0.56)	
> 6 PCPs	186 (62)	185 (61)	62 (61)	0.92 (-0.68, 2.51)		-1.84 (-4.06, 0.37)	
Baseline GAD-7					0.335		0.259
< 15	200 (66)	187 (62)	60 (59)	0.87 (-0.69, 2.43)		-3.32 (-5.55, -1.10)	
>= 15	102 (34)	114 (38)	41 (41)	-0.09 (-2.17, 1.99)		-1.12 (-3.87, 1.62)	
Baseline PHQ-9					0.224		0.264
< 15	180 (60)	182 (60)	60 (60)	0.56 (-1.03, 2.16)		-2.02 (-4.21, 0.18)	
>= 15	122 (40)	119 (40)	40 (40)	0.66 (-1.34, 2.65)		-3.23 (-6.03, -0.44)	
Living Alone					0.186		0.008
No	242 (80)	247 (82)	90 (89)	0.63 (-0.76, 2.01)		-2.11 (-3.95, -0.26)	
Yes	60 (20)	54 (18)	11 (11)	0.3 (-2.54, 3.14)		-5.87 (-10.97, -0.77)	

Cohen's d (95%CI)

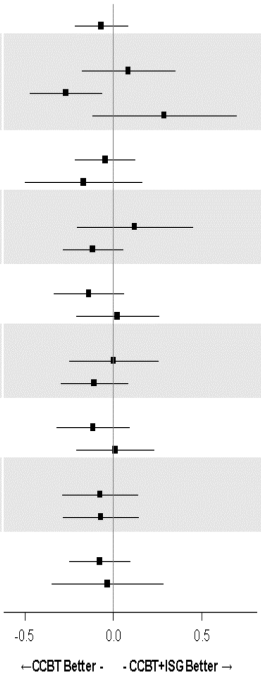

Cohen's d (95%CI)

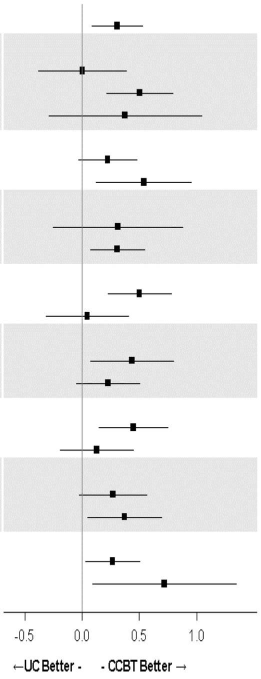

Subgroup	# Patients (%) CCBT+ISG	CCBT	UC	Between Group Diff (95%CI) CCBT+ISG vs CCBT	P-Value	Between Group Diff (95%CI) CCBT vs UC	P-Value
Overall	302	301	101	0.11 (-1.26, 1.49)	0.872	-2.3 (-4.21, -0.4)	0.018
Age					0.007		0.006
18 - 34 yr	111 (37)	108 (36)	37 (37)	-1.50 (-3.79, 0.79)		-1.25 (-4.38, 1.87)	
35 - 59 yr	143 (47)	149 (50)	51 (50)	2.02 (0.07, 3.97)		-3.59 (-6.26, -0.92)	
60 - 75 yr	48 (16)	44 (15)	13 (13)	-2.15 (-5.55, 1.25)		-1.02 (-6.14, 4.09)	
Race					0.445		0.083
White	242 (80)	257 (85)	77 (76)	0.08 (-1.43, 1.6)		-1.44 (-3.61, 0.73)	
Non-White	60 (20)	44 (15)	24 (24)	0.58 (-2.68, 3.83)		-5.44 (-9.49, -1.38)	
Gender					0.326		0.719
Male	57 (19)	66 (22)	19 (19)	-1.32 (-4.34, 1.71)		-0.1 (-4.5, 4.3)	
Female	245 (81)	235 (78)	82 (81)	0.46 (-1.08, 2)		-2.79 (-4.9, -0.68)	
Education					0.710		0.193
< 4 yr college degree	158 (52)	164 (54)	49 (49)	0.34 (-1.54, 2.22)		-4.06 (-6.73, -1.39)	
>= 4 yr college degree	144 (48)	137 (46)	52 (51)	-0.19 (-2.2, 1.82)		-0.43 (-3.14, 2.28)	
Practice Size					0.424		0.317
<= 6 PCPs	116 (38)	116 (39)	39 (39)	-0.18 (-2.39, 2.03)		-3.25 (-6.3, -0.2)	
> 6 PCPs	186 (62)	185 (61)	62 (61)	0.3 (-1.45, 2.05)		-1.69 (-4.12, 0.74)	
Baseline GAD-7					0.092		0.346
< 15	200 (66)	187 (62)	60 (59)	0.47 (-1.23, 2.18)		-3.56 (-5.99, -1.12)	
>= 15	102 (34)	114 (38)	41 (41)	-0.7 (-2.97, 1.58)		-0.52 (-3.53, 2.49)	
Baseline PHQ-9					0.363		0.978
< 15	180 (60)	182 (60)	60 (60)	0.21 (-1.54, 1.97)		-2.27 (-4.69, 0.15)	
>= 15	122 (40)	119 (40)	40 (40)	0.05 (-2.14, 2.25)		-2.52 (-5.6, 0.56)	
Living Alone					0.103		0.010
No	242 (80)	247 (82)	90 (89)	-0.26 (-1.78, 1.26)		-1.84 (-3.87, 0.19)	
Yes	60 (20)	54 (18)	11 (11)	1.61 (-1.51, 4.73)		-6.65 (-12.25, -1.05)	

Cohen's d (95%CI)

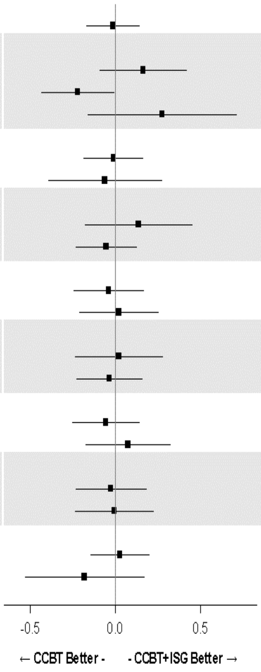

Cohen's d (95%CI)

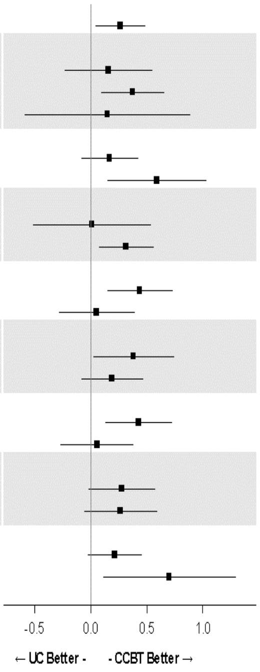

© 2017 Rollman BL et al. *JAMA Psychiatry.*
